# A new inhibitor of the β-arrestin/AP2 endocytic complex reveals interplay between GPCR internalization and signalling

**DOI:** 10.1038/ncomms15054

**Published:** 2017-04-18

**Authors:** Alexandre Beautrait, Justine S. Paradis, Brandon Zimmerman, Jenna Giubilaro, Ljiljana Nikolajev, Sylvain Armando, Hiroyuki Kobayashi, Lama Yamani, Yoon Namkung, Franziska M. Heydenreich, Etienne Khoury, Martin Audet, Philippe P. Roux, Dmitry B. Veprintsev, Stéphane A. Laporte, Michel Bouvier

**Affiliations:** 1 Department of Biochemistry, Institute for Research in Immunology and Cancer (IRIC), Université de Montréal, Montréal, Quebec, Canada H3T 1J4; 2 Department of Molecular Biology Program, Institute for Research in Immunology and Cancer (IRIC), Université de Montréal, Montréal, Quebec, Canada H3T 1J4; 3 Department of Pharmacology and Therapeutics, Research Institute of McGill University Health Centre (RI-MUHC), McGill University, Montréal, Quebec, Canada H4A 3J1; 4 Department of Medicine, Research Institute of McGill University Health Centre (RI-MUHC), McGill University, Montréal, Quebec, Canada H4A 3J1; 5 Laboratory of Biomolecular Research, Department of Biology and Chemistry, Paul Scherrer Institut,5232 Villigen PSI, Switzerland, Switzerland; 6 Department of Pathology and Cellular Biology, Institute of Research in Immunology and Cancer (IRIC), Université de Montréal, Montréal, Quebec, Canada H3T 1J4; 7 Department of Anatomy and Cell Biology, Research Institute of McGill University Health Centre (RI-MUHC), McGill University, Montréal, Quebec, Canada H4A 3J1

## Abstract

In addition to G protein-coupled receptor (GPCR) desensitization and endocytosis, β-arrestin recruitment to ligand-stimulated GPCRs promotes non-canonical signalling cascades. Distinguishing the respective contributions of β-arrestin recruitment to the receptor and β-arrestin-promoted endocytosis in propagating receptor signalling has been limited by the lack of selective analytical tools. Here, using a combination of virtual screening and cell-based assays, we have identified a small molecule that selectively inhibits the interaction between β-arrestin and the β2-adaptin subunit of the clathrin adaptor protein AP2 without interfering with the formation of receptor/β-arrestin complexes. This selective β-arrestin/β2-adaptin inhibitor (Barbadin) blocks agonist-promoted endocytosis of the prototypical β2-adrenergic (β2AR), V2-vasopressin (V2R) and angiotensin-II type-1 (AT1R) receptors, but does not affect β-arrestin-independent (transferrin) or AP2-independent (endothelin-A) receptor internalization. Interestingly, Barbadin fully blocks V2R-stimulated ERK1/2 activation and blunts cAMP accumulation promoted by both V2R and β2AR, supporting the concept of β-arrestin/AP2-dependent signalling for both G protein-dependent and -independent pathways.

## Abstract (web summary)

Beta-arrestins play central roles in the mechanisms regulating GPCR signalling and trafficking. Here the authors identify a selective inhibitor of the interaction between β-arrestin and the β2-adaptin subunit of the clathrin adaptor protein AP-2, which they use to dissect the role of the β-arrestin/β2-adaptin interaction in GPCR signalling.

β-arrestins (β-arrestin1 and β-arrestin2) play central roles in the mechanisms regulating G protein-coupled receptor (GPCR) signalling and trafficking^1^,^2^. The recruitment of β-arrestin to phosphorylated activated GPCRs at the plasma membrane, following sustained agonist stimulation, promotes desensitization by functionally uncoupling the activated receptors from their heterotrimeric G proteins^3^. Complexes formed between ligand-occupied GPCRs and β-arrestin are then directed to the endocytic machinery, leading to their interaction with clathrin and its adaptor protein AP2, followed by the internalization of the receptors^4^,^5^,^6^. Once internalized and targeted to early endosomes, receptors can either be recycled back to the plasma membrane or targeted toward lysosomal degradation. The fate of the endocytosed receptors has been inferred to depend largely on the stability of the interaction between the internalized receptor and β-arrestin^7^; where the more labile complexes lead to rapid recycling of receptor, whereas more stable interactions prevent recycling and favour receptor degradation. Although endocytosis was first linked to receptor desensitization and resensitization, more recent evidence suggest that internalized receptors can engage in various signalling activities^8^,^9^. For instance, endocytosis has been linked to the β-arrestin-dependent activation of mitogen-activated protein kinases (MAPK)^10^ and the sustained activation of adenylyl-cyclase by some receptors^11^,^12^,^13^. Despite the well-established contribution of β-arrestins in various steps of receptor trafficking and signalling, spatial and temporal causative links between these responses have remained difficult to probe, mainly because of the lack of selective pharmacological tools.

Several endocytic paths have been described for GPCRs, including both clathrin-dependent and -independent events^14^,^15^, with the vast majority of GPCRs undergoing β-arrestin-dependent, clathrin-mediated endocytosis. Upon binding to agonist-bound receptor, β-arrestin undergoes a major conformational change, adopting an active conformation that is characterized by the release of its C-terminus, which is buried in its polar core^16^. The exposed β-arrestin C-tail constitutes an accessible site (DxxFxxFxxxR motif) for the binding of the β2-adaptin subunit of the AP2 complex, which directs the receptor/β-arrestin complexes into maturing clathrin-coated pits (CCPs)^17^,^18^. A tripartite interaction exists between β-arrestin, AP2 and clathrin in CCPs (that is, each protein involved in a direct interaction with the other two proteins) and the interaction between β-arrestin and AP2 is an important initial step in localizing GPCRs into the CCP, following agonist stimulation^17^. The structural determinants for β-arrestin and AP2 interaction have been well-defined^17^,^19^, and more recently validated by a co-crystal between the β2-adaptin subunit of AP2 and a peptide fragment of β-arrestin1 C-terminus^20^. Most of our knowledge concerning GPCR endocytosis and β-arrestin-dependent signalling has been gained through mutagenesis and protein depletion studies in particular for β-arrestins, which would indiscriminately affect recruitment of β-arrestin to receptors, desensitization, endocytosis and β-arrestin-mediated signalling^21^,^22^,^23^. Specific pharmacological probes targeting β-arrestin/AP2 complexes would be invaluable for determining the role of this complex and clathrin-mediated endocytosis in GPCR signalling.

In this light, we have used a virtual screening approach to identify specific inhibitors of β-arrestin/AP2 complexes. For this purpose, we took advantage of a well-defined groove within the β2-adaptin ear platform subdomain, which accommodates the carboxyl-tail of β-arrestin^20^, hence providing a putative site for a small molecule interfering with the β-arrestin/AP2 interaction. On the basis of this structural insight, we combined virtual screening with a sensitive bioluminescence resonance energy transfer (BRET)-based assay^24^ to identify and validate novel inhibitors of the β-arrestin/β2-adaptin interaction. From five compounds found to modulate the β-arrestin/β2-adaptin interaction, two were inhibitors and one of them, which we named β-arrestin/β2-adaptin interaction inhibitor (Barbadin), was further characterized and used as a tool to study the interplay between receptor GPCR internalization and signalling.

## Results

### Screening for a β-arrestin/β2-adaptin interaction inhibitor

**Figure 1:**
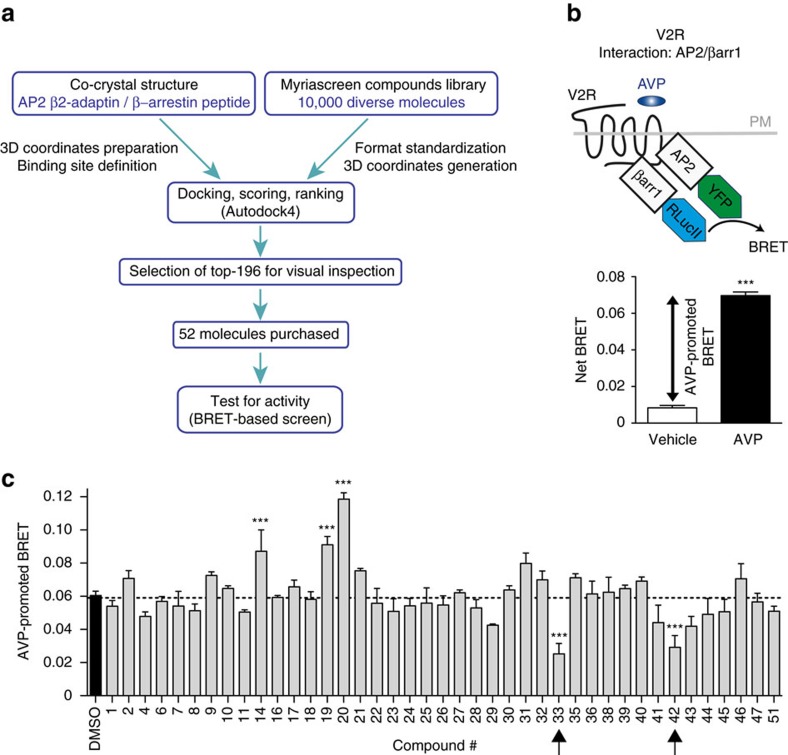
Screen for inhibitors of the interaction between β2-adaptin and β-arrestin. (**a**) Flowchart outlining the steps of the virtual screening and the selection of candidate molecules to be tested. (**b**) Schematic representation and dynamic range of the BRET-based assay used to test the compounds selected from the virtual screen that monitors the β2-adaptin/β-arrestin1 interaction. HEK293T cells were transfected with β2-adaptin-YFP, β-arrestin1-RlucII and myc-V2R. BRET was measured following V2R stimulation with 100 nM AVP for 45 min. Data are the mean±s.e.m. of three independent experiments. Statistical analysis was performed using a paired Student's test (***P<0.001). (**c**) β2-adaptin/β-arrestin1 interaction was assessed as described in (**b**) with AVP-stimulation following pre-incubation with the indicated compounds (100 μM) for 25 min. Data are the mean±s.e.m. of three independent experiments. One-way ANOVA followed by Tuckey's post-hoc tests were used to assess the statistical significance of the compound-induced BRET modulation compared to DMSO (***P<0.001).

To identify candidate inhibitors of the β-arrestin/β2-adaptin interaction, we first performed a structure-based virtual screen through the use of molecular docking calculations (Fig. 1a, and methods). We took advantage of the available coordinates of the crystal structure of a complex between the β2-adaptin ear domain of the clathrin adaptor protein AP2 and a C-terminal peptide of β-arrestin1 (PDB entry 2IV8) (ref. 20). In this complex, the β-arrestin peptide is situated in a groove located in the β2-adaptin ear platform subdomain, where Phe-388, Phe-391 and Arg-395 from β-arrestin1 interact with key Glu-849, Tyr-888 and Glu-902 residues of β2-adaptin. The virtual screen was performed using a region of the β2-adaptin ear platform that included the entire binding groove as the target, and the Myriascreen diversity collection of 10,000 compounds selected for their structural diversity and drug-likeness from an original chemical library of 300,000 molecules. Subsequently to docking calculations, the poses of the best-ranked molecules based on predicted binding energy (196 compounds) were visually inspected to select the candidates that make favourable interactions with residues from the β2-adaptin groove, reported to be critical for β-arrestin binding^19^. Among these best candidates, 52 compounds were selected based on availability criteria. The list of these compounds with their acquisition number is presented in Supplementary Table 1.

To assess the ability of the 52 compounds to affect the β-arrestin/β2-adaptin interaction, we used a BRET-based assay that directly monitors the interaction between these two proteins following agonist-stimulation of GPCRs (ref. 24). The vasopressin type-2 receptor (V2R), that was previously shown to promote a large BRET increase between β-arrestin1-RlucII and β2-adaptin-YFP upon arginine vasopressin (AVP) stimulation^24^ (Fig. 1b), was chosen as receptor model for the screen. Before assessing the ability of the 52 compounds to modulate the BRET between β2-adaptin-YFP and β-arrestin1-RlucII, we verified whether the molecules could spectrally interfere with BRET measurements. When tested in HEK293 cells overexpressing either the β-arrestin1-RlucII or the β2-adaptin-YFP alone, 11 of the 52 compounds that had a significant effect on either luminescence or fluorescence signals were discarded (Supplementary Fig. 1). The remaining 41 compounds were tested in live cells, using the aforementioned BRET-based assay in which the V2R, β2-adaptin-YFP and β-arrestin1-RlucII were co-expressed. As shown in Fig. 1c, five compounds significantly modulated the AVP-promoted BRET. Compounds #14, #19 and #20 potentiated, whereas compounds #33 and #42 inhibited the AVP-promoted BRET between β2-adaptin-YFP and β-arrestin1-RlucII. Compounds #14, #19 and #20 might favour the interaction between β-arrestin1 and β2-adaptin, or modify the complex in a way that brings the YFP and RlucII moieties closer, or in an orientation which is more permissive for energy transfer. Although they may be interesting molecules in their own rights, we focused on characterizing compounds #33 and #42 because we sought to identify inhibitors of the β2-adaptin/β-arrestin complex.

**Figure 2:**
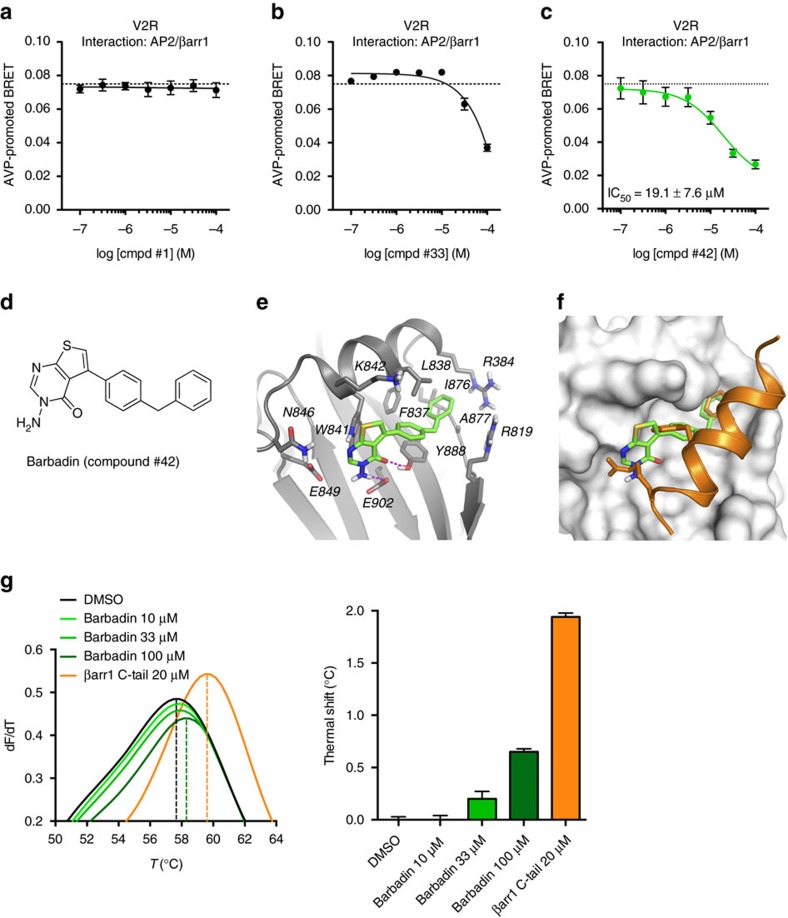
Identification of Barbadin (compound #42) as an inhibitor of the interaction between β2-adaptin and β-arrestin. (**a**–**c**) Concentration-response curves of compounds #1, #33 and #42 selected from the BRET-based screen (Fig. 1) using the same β2-adaptin/β-arrestin1 interaction assay. Dotted line represents the level of AVP-promoted BRET upon pre-incubation with DMSO. Data are the mean±s.e.m. of three independent experiments. (**d**) Chemical structure of Barbadin (IUPAC name: 3-amino-5-(4-benzylphenyl)-3H,4H-thieno[2,3-d]pyrimidin-4-one). (**e**,**f**) Docking pose of Barbadin (green sticks) within the groove of β2-adaptin platform subdomain (grey ribbon (**e**), or *surface* (**f**)) that is the site of interaction with β-arrestin (orange ribbon and sticks (**f**)). β2-adaptin residues known to interact with β-arrestin are labelled and shown as grey stick. Hydrogen-bonds interactions between Barbadin and both Tyr-888 and Glu-902 are depicted as magenta dotted lines (**e**). Superimposition of Barbadin with the β-arrestin1 C-terminus peptide as in the co-crystal structure (PDB entry 2IV8), where Phe-388, Phe-391 and Arg-395 are the three key residues for β-arrestin binding interaction (shown from top to bottom as orange sticks) (**f**). (**g**) Thermal denaturation of β2-adaptin (residues 700–937) and concentration-dependent stabilization effect of Barbadin. The maximum of the first derivatives of fluorescence (dF/dT) data from differential scanning fluorimetry corresponds to the melting temperatures (Tm, indicated with a dotted line) of β2-adaptin in the presence of DMSO, Barbadin or β-arrestin1 C-tail peptide (positive control) at the indicated concentrations.

To assess the potency of these compounds, concentration-response curves of the inhibitory actions of the compounds on the V2R-stimulated β-arrestin/β2-adaptin interaction were carried out. In addition to compounds #33 and #42, compound #1 was included in the analysis as a negative control (Fig. 2a–c). As expected, #1 was completely inactive even at the highest concentration used (Fig. 2a), whereas both #33 and #42 showed concentration-dependent inhibitory responses. While #33 had a low-potency (>50 μM) that was difficult to estimate with precision due to incomplete inhibition curve (Fig. 2b), #42 showed a well-defined sigmoidal inhibitory curve yielding an estimated IC_50_ of 19.1 μM for β-arrestin1 (Fig. 2c) and 15.6 μM for β-arrestin2 (Supplementary Fig. 2a). Compound #42, which structure is shown in Fig. 2d, represented a validated hit for a potential inhibitor of the complex and hence named Barbadin, and further characterized at the expense of #33 that showed lower potency. First, the reversible mode of action of Barbadin was assessed by incubating cells with Barbadin for 30 min and monitoring BRET between β2-adaptin and β-arrestin1 following, or not, extensive wash before AVP stimulation. As shown in Supplementary Fig. 2b, the washing procedure completely prevented the action of Barbadin demonstrating its reversibility.

To further investigate the potential binding mode of Barbadin on AP2, we examined the docking poses obtained for this compound. The docking calculations resulted in a single best pose, based on its prevalence among the possible solutions and the lowest binding energy that positioned Barbadin within the groove of the β2-adaptin platform subdomain (Fig. 2e). The benzylphenyl moiety of Barbadin tightly fits a hydrophobic pocket lined mainly by Phe-837, Leu-838, Ile-876, Ala-877 and Tyr-888, via a set of non-polar and π–π aromatic interactions. Superimposition of the docking pose of Barbadin with the β2-adaptin/β-arrestin C-tail co-crystal structure suggests that both phenyl rings of Barbadin are adopting very similar binding modes as both β-arrestin's key Phe-391 and Phe-395 residues (Fig. 2f). The thieno-pyrimidinone moiety interacts through π–π aromatic interactions with Trp-841, and also through a network of two hydrogen bonds involving Tyr-888 and Glu-902 (Fig. 2e), two residues of β2-adaptin shown to be critical for the binding of β-arrestin^19^. To investigate the role of the benzylphenyl moiety in Barbadin's inhibitory activity, we conducted a preliminary structure-activity relationship study with four analogues of Barbadin. As shown in Supplementary Fig. 3, a branched aliphatic and/or extended phenyl group on Barbadin's thieno-pyrimidinone core is essential for its inhibitory action, consistent with the docking pose.

In addition, to assess the direct binding of Barbadin to β2-adaptin we performed a thermal-shift assay using purified appendage domain of β2-adaptin (β2-appendage, amino-acids with sequence: 700–937). The stability curves obtained in Fig. 2g show that, similarly to a 20-mer β-arrestin C-tail peptide known to bind to the β-appendage^20^, Barbadin promoted a dose-dependent rightward shift of the melting temperature consistent with a stabilization of the β-appendage due to the direct binding of the compound.

### Barbadin selectively inhibits the β-arrestin/AP2 interaction

**Figure 3:**
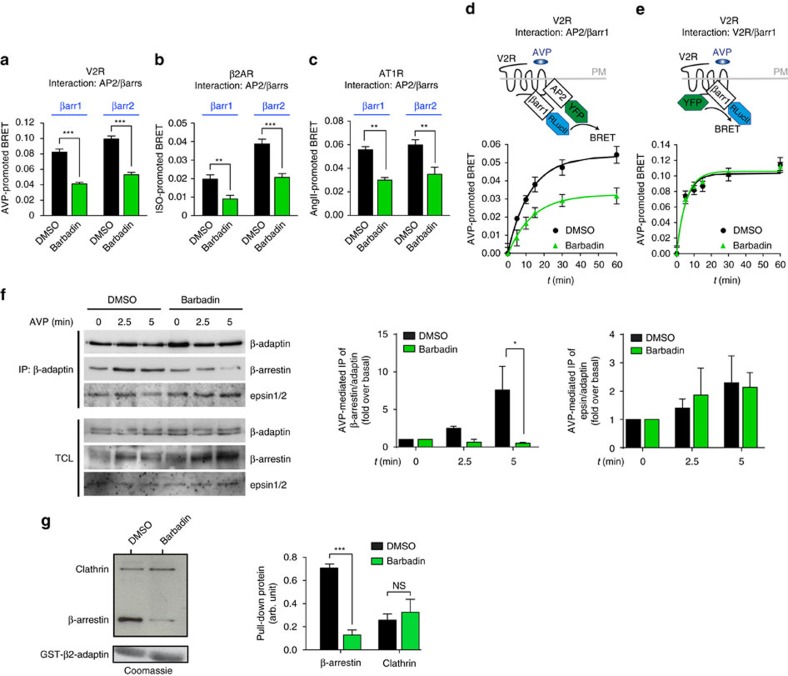
Barbadin specifically blocks the interaction between β2-adaptin and β-arrestin. (**a**–**c**) BRET-based assay monitoring the interaction between β2-adaptin-YFP and either β-arrestin1-RlucII or β-arrestin2-RlucII. HEK293T cells were pre-incubated with DMSO or Barbadin (100 μM) for 30 min before 45 min receptor stimulation with AVP (100 nM, **a**), ISO (10 μM, **b**) or AngII (100 nM, **c**). Data are the mean±s.e.m. of at least three independent experiments and unpaired *t*-test were used to assess statistical significance (***P<0.001; **P<0.005). (**d**,**e**) BRET-based kinetics monitoring the interaction between β-arrestin1-RlucII and β2-adaptin-YFP (**d**) or V2R-YFP (**e**) in HEK293T cells pretreated with DMSO or Barbadin (100 μM) for 30 min before receptor stimulation with AVP (100 nM) for the indicated times. Data are the mean±s.e.m. of three independent experiments. (**f**) Effect of Barbadin on the co-immunoprecipitation between β-arrestins and AP2. HEK293SL cells expressing V2R and Flag-β-arrestin2 were pretreated for 20 min with DMSO or Barbadin (50 μM) before stimulation by AVP (1 μM) for 2.5 or 5 min. Endogenous AP2 complexes (using the AP1/2 antibody) were immunoprecipitated (IP) from total cell lysates (TCL), and then analysed by western blot using anti-Flag, anti-epsin or anti-adaptin antibodies as described in the Material and Methods. TCLs represent 5% of input used for IP. IP were quantified over three independent experiments and statistical significance was assessed by a two-way ANOVA followed by Bonferroni's post-hoc tests (*P<0.05). (**g**) Effects of Barbadin on the pull-down of β-arrestin1 and clathrin with GST-β2-adaptin. DMSO or Barbadin (100 μM) were incubated with GST-β2-adaptin (592–937) beads. HEK293T cells transfected with Flag-β-arrestin1 were lysed and added to the beads. The amounts of GST-β2-adaptin were detected by Coomassie, whereas β-arrestin1 and clathrin associated with GST-β2-adaptin were detected by western blot using anti-Flag and anti-clathrin (heavy chain) antibodies, respectively. Relative intensities were normalized to the GST input for each condition and densitometry data are the mean±s.e.m. of three independent experiments and analysed using a two-way ANOVA followed by Bonferroni's post-hoc tests (NS, non-significant; ***P<0.001).

To rule out the possibility that Barbadin interferes directly at the level of the V2R, we assessed its effect on the β-arrestin1/β2-adaptin interaction by BRET, promoted by two additional GPCRs, the β2-adrenergic receptor (β2AR) and the angiotensin-II type-1 receptor (AT1R) (Fig. 3a–c). As was the case for the V2R, Barbadin inhibited the BRET between β-arrestin1-RlucII and β2-adaptin-YFP promoted by the activation of both receptors. The β2AR was selected because it differs from the V2R and AT1R in its mode of interaction with the β-arrestins^7^. The β2AR is classified as a class A receptor, interacting transiently with β-arrestins and rapidly recycling back from the endosomes to the cell surface, whereas V2R and AT1R are class B receptors forming tighter receptor/β-arrestin complexes that reside for longer times in endosomes before being targeted to lysosomal degradation. These observations strongly suggest that the inhibition occurs at the level of the β-arrestin/β2-adaptin interaction and is not a receptor specific effect.

We next evaluated the mode of action of Barbadin by testing its effect on the other isoform of β-arrestin, β-arrestin2, that is highly homologous to β-arrestin1 and would be predicted to interact with β2-adaptin in a similar manner. As shown in Fig. 3a–c, Barbadin significantly inhibited the interaction between β2-adaptin and both β-arrestins after stimulation of V2R, β2AR or AT1R. Moreover, the extent of inhibition was comparable, in all cases Barbadin inhibiting the BRET signal by ≈50%. The higher agonist-promoted signal observed for β-arrestin2 versus β-arrestin1 upon β2AR stimulation, compared to the similar signals measured for both β-arrestin isoforms following V2R or AT1R stimulation, is consistent with the notion that class A, but not class B receptors bind β-arrestin2 with greater avidity than β-arrestin1 (refs 7, 24).

To further assess whether Barbadin acts by selectively inhibiting the interaction between β2-adaptin and β-arrestin, and not by inhibiting the recruitment of β-arrestin to GPCRs, the effect of Barbadin was also tested on the time-dependent AVP-promoted BRET between either β2-adaptin-YFP and β-arrestin1-RlucII or V2R-YFP and β-arrestin1-RlucII (Fig. 3d,e). Barbadin strongly inhibited AVP-promoted BRET between β-arrestin1 and β2-adaptin but was without effect on the V2R/β-arrestin1 BRET induced upon AVP stimulation, thus confirming a selective action on the β-arrestin/β2-adaptin complex. Similar results were obtained for the class A β2AR (Supplementary Fig. 4a,b).

Fluorescence confocal microscopy confirmed that Barbadin did not inhibit the recruitment of β-arrestin2 to the plasma membrane upon V2R activation (Supplementary Fig. 4c). In fact, co-localization of V2R and β-arrestin2 at the plasma membrane was more readily detected following Barbadin treatment as compared to control conditions, consistent with the notion that it does not affect receptor/β-arrestin interaction but that it inhibits internalization of receptor/β-arrestin complexes, hence increasing their accumulation at the plasma membrane.

The specificity of the effect of Barbadin on the endocytic complex formation was also validated by co-immunoprecipitation and GST pull-down experiments. Figure 3f illustrates that Barbadin significantly inhibited AVP-promoted co-immunoprecipitation between endogenous β-adaptins and β-arrestin1/2 but did not affect the association with epsin, a protein interacting with lower affinity than β-arrestin at a different site on β2-adaptin's ear platform subdomain^25^. Similarly, Barbadin significantly inhibited the ability of GST-β2-adaptin to pull-down β-arrestin1 (Fig. 3g) but had no effect on the pull-down of clathrin, which is known to interact at β2-adaptin sites (the hinge region and the ear β-sandwich subdomain) that are distinct from the interaction site of β-arrestin. Taken together, these results indicate that Barbadin selectively blocks the interaction between β-arrestin and β2-adaptin as predicted by the *in silico* docking and the BRET-based assays.

### Barbadin blocks β-arrestin/AP2-dependent GPCR endocytosis

**Figure 4:**
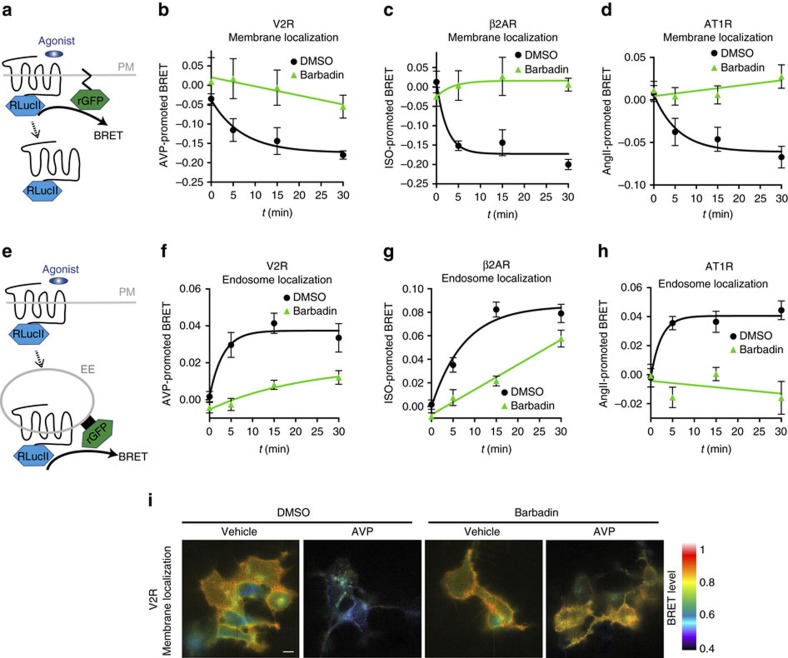
Barbadin inhibits GPCRs endocytosis. (**a**,**e**) Schematic representation of the ebBRET-based assay used to follow agonist-induced receptor loss from the cell surface by monitoring the interaction between receptor-RLucII and rGFP-CAAX (**a**) or its translocation into endosomes using rGFP-FYVE (**e**). (**b**–**d**,**f**–**h**) V2R, β2AR or AT1R interaction with either rGFP-CAAX (**b**–**d**, respectively) or rGFP-FYVE (**f**–**h**, respectively) was assessed by BRET following HEK293T cells pre-incubation with DMSO or Barbadin (100 μM) for 30 min before AVP (100 nM), ISO (10 μM) or AngII (1 μM) stimulation for the indicated times. Data are the mean±s.e.m. of a least three independent experiments. (**i**) V2R localization was imaged by BRET. HEK293T cells were transfected with V2R-RlucII and rGFP-CAAX, pretreated with DMSO or Barbadin (100 μM) for 30 min and then stimulated with 100 nM AVP for 30 min. To generate BRET images, the ratio of acceptor photon counts to donor photon counts was calculated for each pixel and expressed as a colour-coded heat map (lowest being black and purple, and highest red and white). Scale bar, 10 μm.

Because the interaction between β-arrestins and β2-adaptin is important for initiating receptor internalization, we next sought to determine whether Barbadin could be used as a pharmacological tool, to block the ligand-promoted endocytosis of GPCRs. For this purpose, we used enhanced bystander BRET (ebBRET) trafficking sensors allowing to monitor both receptor disappearance from the plasma membrane (rGFP-CAAX sensor, Fig. 4a) and their accumulation in early endosomes (rGFP-FYVE, Fig. 4e) in spectrometric and microscopy-based assays^26^. As shown in Fig. 4a–h, Barbadin blocked the time-dependent agonist-induced loss of V2R, β2AR and AT1R from the cell surface (Fig. 4b–d), and consequently prevented their appearance into early endosomes (Fig. 4f–h), demonstrating that it acts as an efficient endocytosis inhibitor. Notably, the IC_50_ for the inhibitory action of Barbadin on endocytosis are similar to those observed for its action on the β-arrestin/AP2 interaction (Fig. 2c and Supplementary Fig. 2a), consistent with its mode of action (Supplementary Fig. 5a–h). The effect of Barbadin on endocytosis was further confirmed using BRET-microscopy allowing to image receptor localization at the plasma membrane (Fig. 4i and Supplementary Fig. 5i), and in early endosomes (Supplementary Fig. 5j), coherent with the spectrometric measurements (Fig. 4b–d,f–h).

**Figure 5:**
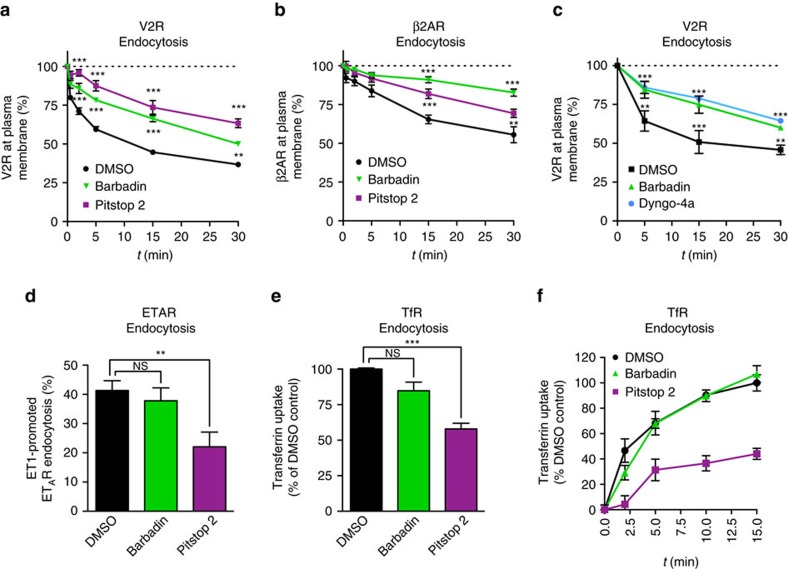
Barbadin inhibits the β-arrestin- and AP2-dependent endocytosis of GPCRs. (**a**–**c**) Cell surface expression of HA-V2R-Venus (**a**,**c**) or HA-β2AR-Venus (**b**) transfected in HEK293T cells was monitored by FACS following pre-incubation with DMSO, Barbadin (100 μM), Pitstop2 (100 μM) or Dyngo-4a (30 μM) for 30 min, before agonist stimulation (AVP (100 nM, **a**,**c**), ISO (10 μM, **b**)) at the indicated times. Data are the mean±s.e.m. of at least three independent experiments. Statistical significance of the effect of Barbadin, Pitstop 2 and Dyngo, as compared to DMSO, was assessed by a two-way ANOVA followed by Bonferroni's post-hoc tests (**P<0.01; ***P<0.001). (**d**) Cell surface expression of HA-ET_A_R was monitored by FACS following pre-incubation with DMSO, Barbadin (100 μM) or Pitstop2 (100 μM) for 30 min, before ET1 (10 nM) stimulation for 30 min. Data are the mean±s.e.m. of at least three independent experiments. Statistical significance was assessed by a one-way ANOVA followed by Tuckey's post-hoc tests (NS, non-significant; **P<0.01). (**e**,**f**) Native transferrin receptor (TfR) uptake was monitored by FACS in HeLa cells. Cells were pretreated with DMSO, Barbadin (100 μM), or Pitstop2 (100 μM) for 30 min, then incubated with Alexa Fluor 633-conjugated transferrin antibody (100 μg ml^−1^) for 30 min at 4 °C, and finally shifted to 37 °C for 15 min (**e**) or the indicated time (**f**). Data are the mean±s.e.m. of three independent experiments. Statistical significance was assessed by a one-way ANOVA followed by Tuckey's post-hoc tests (NS, non-significant; **P<0.01; ***P<0.001).

To orthogonally validate the BRET-based endocytosis measurements, we used flow-cytometry (FACS) with double-tagged receptors where a HA epitope and a Venus fluorescent protein are fused to amino- and carboxy-termini, respectively, allowing to monitor both cell surface (HA immunofluorescence) and total receptor (venus fluorescence) expression^27^. As shown in Fig. 5a–c, agonist stimulation of cells expressing either the V2R or β2AR led to a time-dependent disappearance of cell surface signals corresponding to agonist-promoted endocytosis. Barbadin significantly inhibited FACS-monitored endocytosis to extents comparable to Pitstop2 and Dyngo-4a, two known inhibitors of both clathrin-dependent and independent endocytosis^28^,^29^,^30^ (Fig. 5a–c). Since both β2AR and V2R are known to undergo β-arrestin and β2-adaptin-dependent endocytosis, the effect of Barbadin on agonist-promoted endocytosis is likely explained by the inhibitory action of Barbadin on the β-arrestin/β2-adaptin interaction. To further validate this mode of action, we used the type A endothelin receptor (ET_A_R) that undergoes β-arrestin-dependent but clathrin-independent endocytosis into caveolae^31^, and which involves the recruitment of β-arrestin to the receptor without engagement of AP2 (ref. 24). As predicted from its proposed mode of action, Barbadin did not inhibit endothelin-promoted ET_A_R endocytosis (Fig. 5d). In contrast, Pitstop2 significantly blocked agonist-stimulated ET_A_R endocytosis consistent with its clathrin-independent action^29^. To further assess Barbadin selectivity, we tested its effect on transferrin endocytosis occurring through clathrin-coated pits and involving the transferrin receptor (TfR), a non-GPCR that interacts directly with AP2 independently of β-arrestin^32^. βarbadin did not significantly inhibit the internalization of transferrin at any of the time points examined, whereas it was blocked by Pitstop2 as early as 2 min after stimulation (Fig. 5e,f). The ability of Barbadin to block the endocytosis of natively expressed GPCR was also confirmed by monitoring the agonist-promoted loss of AT1R from cell surface using radio-ligand binding in vascular smooth muscle cells (Supplementary Fig. 6). Taken together, these results indicate that Barbadin is a suitable tool to study β-arrestin/AP2-dependent endocytosis.

**Figure 6:**
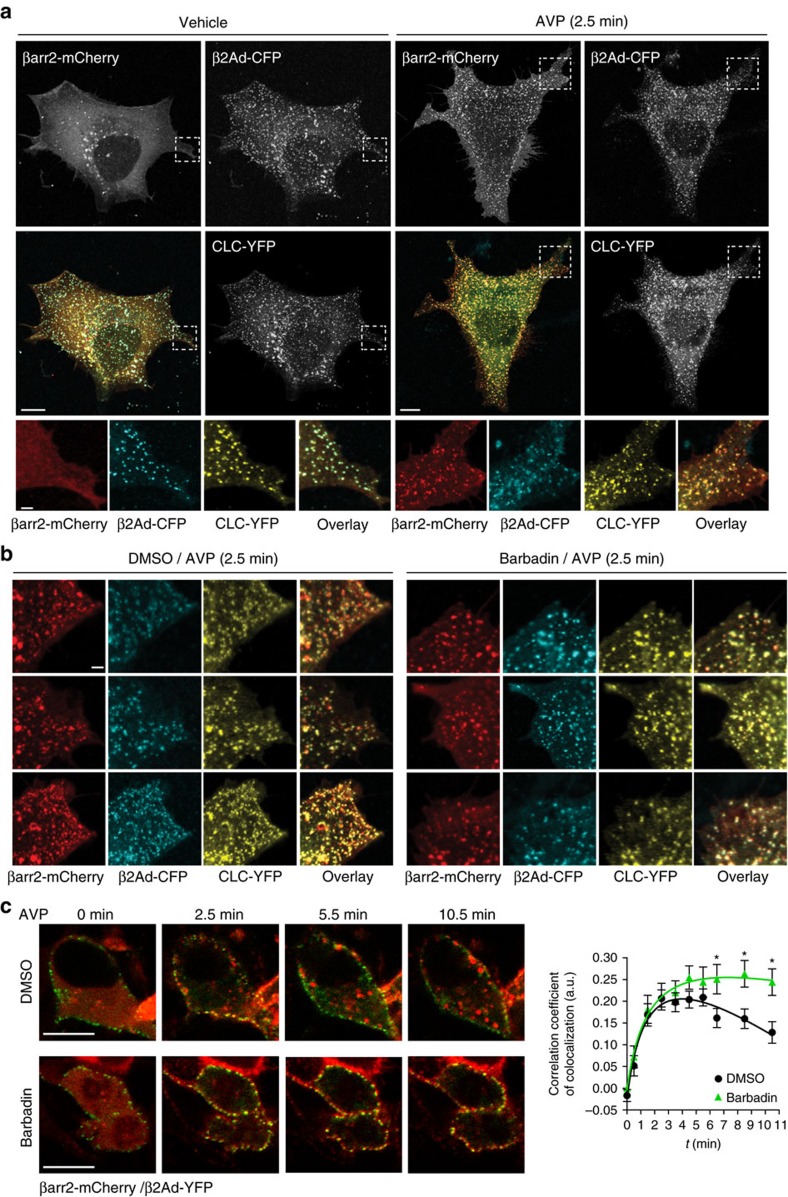
Barbadin induces the retention of receptor/β-arrestin complexes in clathrin-coated pits (CCPs) at the membrane. (**a**,**b**) Confocal images and colocalization of β-arrestin2, β2-adaptin, and clathrin light chain-(CLC) in CCPs from AVP-stimulated cells expressing V2R. β2-adaptin depleted HEK293 cells (CRISPR-β2Ad-LY5) were transfected with β-arrestin2-mCherry, β2-adaptin-CFP and CLC-YFP, and HA-V2R, and serum-starved for 30 min in the absence or presence of Barbadin (10 μM), before being either left non-stimulated (vehicle) or stimulated with AVP (1 μM) for 2.5 min. Cells were then fixed as described in the Methods section before visualization. Shown in the top panels are black and white micrographs of acquired fluorescent signals from the three-tagged proteins in each channel (red, cyan and yellow), and in colour, are the overlay images. Lower colour insets are close-up images from boxed areas. (**b**) Colour images represent individual and overlay fluorescent signals taken from different areas, from cells transfected, treated and stimulated as in **a**. Scale bars in images of whole cells, 10 μm; and insets, 2 μm. Colocalization quantification is presented in Supplementary Fig. 7b–c. (**c**) Colocalization and quantification of β-arrestin2-mCherry and β2-adaptin-YFP in live HEK293T cells stably expressing V2R. Cells were pretreated with DMSO or Barbadin (10 μM) for 30 min before stimulation with AVP (1 μM) for the indicated durations. Co-localization was quantified using the Pearson correlation coefficient over three independent experiments using 33 or 24 cells for DMSO and Barbadin condition, respectively. Statistical significance of the effect of Barbadin as compared to DMSO was assessed by a two-way ANOVA followed by Bonferroni's post-hoc tests (*P<0.05).

To gain insight on the inhibitory properties of Barbadin on clathrin-mediated internalization of GPCRs, we next examined the sub-cellular distribution of β-arrestin, AP2 and clathrin by confocal fluorescence microscopy in V2R-expressing cells (lacking endogenous β2-adaptin to facilitate the incorporation of CCPs-labelled β2-adaptin into AP2 complexes, Supplementary Fig. 7a) transfected with β-arrestin2-mCherry, β2-adaptin-CFP and clathrin-light-chain-YFP. As shown in Fig. 6a, under basal conditions β2-adaptin and clathrin colocalized in CCPs, whereas β-arrestin is diffuse in the cytosol. On AVP stimulation, β-arrestin translocates to CCPs where it colocalizes with β2-adaptin and clathrin (Fig. 6a,b). A pre-treatment of 30 min with Barbadin did not disrupt the CCPs nor prevented the translocation of β-arrestin to pits (Fig. 6b). In fact, Barbadin led to an increase in β-arrestin/β2-adaptin colocalization (Supplementary Fig. 7b,c) that was even more evident upon longer agonist stimulation (Fig. 6c). The maintenance of the CCPs integrity upon Barbadin treatment is consistent with our previous biochemical data showing that Barbadin has no significant effect on the binding of clathrin to β2-adaptin (Fig. 3g). The observation that β-arrestin translocation to the plasma membrane is not inhibited by Barbadin is also in agreement with our BRET data demonstrating that Barbadin does not interfere with the recruitment of β-arrestin to V2R (Fig. 3e) or β2AR (Supplementary Fig. 4b). Although, the lack of obvious inhibition of β-arrestin and β2-adaptin colocalization by Barbadin appears counter-intuitive considering it blocks the interaction between these two proteins, the localization of β-arrestin to the pits does not result exclusively from its interaction with β2-adaptin. Indeed, many other protein–protein interactions have been implicated in stabilizing GPCR/β-arrestin complexes at the PM and into CCPs, including direct binding of β-arrestin's N-terminal domain^33^ to the μ-subunit of AP2 and an indirect clathrin-mediated link between AP2 and β-arrestin's C-terminal domain^34^,^35^. Therefore, the persistence of β-arrestin in CCPs and its colocalization with AP2 in the presence of Barbadin most likely reflects β-arrestin's ability to directly interact with other components of the CCPs, in particular clathrin itself bringing it in close proximity to β2-adaptin, resulting in colocalization, without a direct interaction. To further test this possibility, we took advantage of a mutant form of β-arrestin lacking the major site of interaction with clathrin (β-arrestin2-ΔClath). As shown in Supplementary Fig. 7d, although β-arrestin2-ΔClath was still recruited into CCPs, where it largely colocalized with β2-adaptin upon AVP stimulation, Barbadin treatment resulted in a marked reduction of β-arrestin2-ΔClath clustering in these CCPs as can be seen by its more diffuse distribution at, or near, the PM, and the reduced colocalization of the mutant β-arrestin with β2-adaptin. These findings are consistent with the notion that even though the interaction between β-arrestin and AP2 plays a critical role for clustering receptor/β-arrestin complexes in CCPs, other interactions such as the ones with clathrin are also important, and that inhibiting only one of them is insufficient to prevent its targeting to CCPs. Yet, Barbadin clearly inhibited the traffic of β-arrestin from the plasma membrane to endosomes that is seen upon sustained agonist stimulation of receptor (Fig. 6c). This suggests that destabilizing the interaction between the β2-adaptin and β-arrestin with Barbadin delays CCPs-mediated internalization kinesis.

### Barbadin modulates GPCR signalling

**Figure 7:**
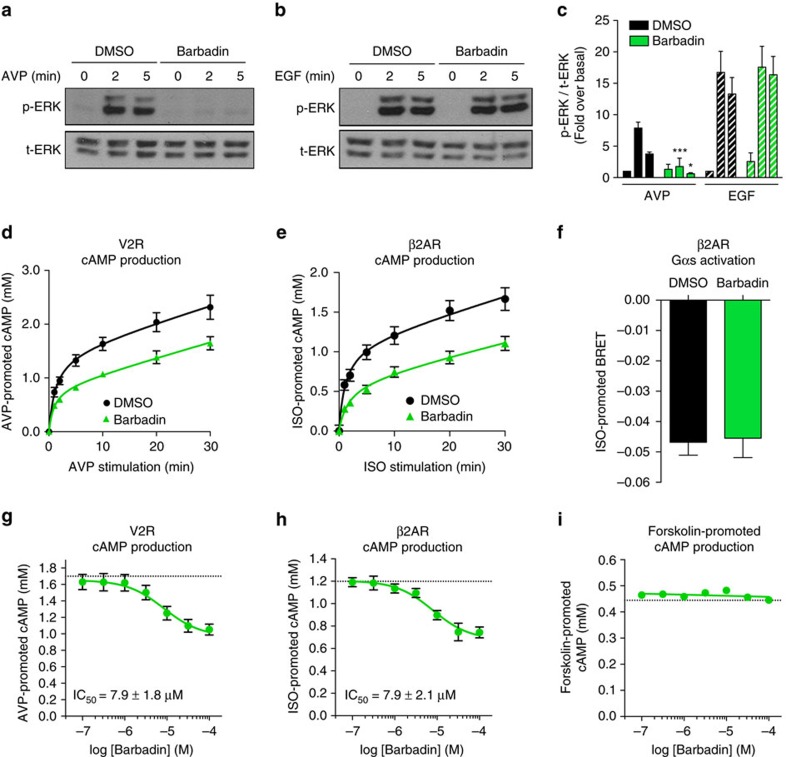
Barbadin inhibits ERK1/2 activation and cAMP accumulation following agonist-stimulation of GPCR. (**a**–**c**) Kinetics of ERK1/2 phosphorylation in HEK293T cells expressing V2R (**a**) or EGFR (**b**) and pretreated with DMSO or Barbadin (50 μM) for 30 min before stimulation with AVP (100 nM, **a**) or EGF (100 ng ml^−1^, **b**) at the indicated times. Western blots were quantified (**c**) and data shown are the mean±s.e.m. of four independent experiments and analysed using a two-way ANOVA followed by Bonferroni's post-tests (*P<0.05; ***P<0.001). (**d**,**e**) Kinetics of the agonist-promoted accumulation of cAMP in HEK293T cells stably expressing V2R (**d**) or β2AR (**e**) and pretreated with DMSO or Barbadin (50 μM) for 30 min before stimulation with AVP (100 nM, **d**) or ISO (10 μM, **e**) at the indicated times. Data are the mean±s.e.m. of three independent experiments. (**f**) ISO-promoted Gs activation measured by BRET in HEK293T cells transfected with HA-β2AR, Gαs-RLucII, Gβ1 and Gγ2-GFP10, pretreated with DMSO or Barbadin (100 μM) for 30 min, before ISO (10 μM) stimulation for 5 min. (**g**,**h**) Concentration-response curves of Barbadin effect on the intracellular cAMP production, in HEK293T cells stably expressing the V2R (**g**) or the β2AR (**h**), upon agonist stimulation for 15 min with AVP (100 nM) or ISO (1 μM), respectively, following pre-treatment with DMSO (grey dotted line) or Barbadin (green) at the indicated concentrations for 30 min. Data are the mean±s.e.m. of three independent experiments. (**i**) Concentration-response curves of Barbadin effect on the intracellular cAMP production, in HEK293T cells stimulated with forskolin (10 μM) for 15 min. Same DMSO and Barbadin pre-treatments as in (**g**,**h**). Data are the mean±s.e.m. of three independent experiments.

Several studies have suggested a role for β-arrestin and/or endocytosis in downstream signalling. Since Barbadin blocks β-arrestin/β2-adaptin interaction and the subsequent endocytosis without affecting the recruitment of β-arrestin to the receptor (Fig. 3d), it represents a unique tool to selectively assess the role of β-arrestin/β2-adaptin-dependent endocytosis in signalling. Given that β-arrestin-mediated endocytosis has been proposed to contribute to the activation of the MAPK by many GPCRs^36^,^37^ including the V2R for which the ERK1/2 activation in HEK293 cells is entirely βarr-dependent^38^, we assessed the effect of Barbadin on the V2R-stimulated ERK1/2 activity. As shown in Fig. 7a,c, Barbadin completely blocked the AVP-stimulated ERK1/2 activation. This effect was selective since Barbadin did not block EGF-stimulated ERK1/2 (Fig. 7b,c). These data indicate that the engagement of β2-adaptin by β-arrestin is essential for the β-arrestin-dependent activation of ERK1/2 by V2R and are consistent with a role for endocytosis in this signalling cascade. When considering the G protein-dependent signalling cascades such as the activation of adenylyl cyclase, the β-arrestin-promoted endocytosis has been proposed to contribute both to the desensitization-resensitization cycle^2^,^39^, as well as sustained endosomal Gs signalling^11^,^12^,^13^. As shown in Fig. 7d,e, Barbadin significantly blunted the time-dependent increase in cyclic AMP (cAMP) production promoted by the agonist stimulation of either V2R or β2AR. This effect was concentration-dependent yielding an IC_50_ of ∼7.9 μM (Fig. 7g,h), which is consistent with the potency of Barbadin to inhibit the β-arrestin/β2-adaptin interaction (Fig. 2c). The selectivity of action is confirmed by the lack of effect of Barbadin on the forskolin-stimulated cAMP production (Fig. 7i) or the activation of Gs as measured by BRET (ref. 40) (Fig. 7f). Such inhibition of the β2AR-stimulated cAMP production could result from the lack of recycling of resensitized receptor to the plasma membrane that normally follows endocytosis. However, the latter explanation is unlikely because, in contrast to β2AR, V2R does not recycle back to the plasma membrane following endocytosis, and therefore this process would not contribute to the inhibition of V2R-stimulated cAMP production observed here. These results therefore indicate, as was recently suggested for some receptors, that inhibition of endocytosis may prevent the adenylyl cyclase signalling that persists following endocytosis^11^,^12^.

## Discussion

The present study led to the identification of the first chemical probe that specifically inhibits the interaction between β-arrestin and the β2-adaptin subunit of AP2. We validated the important role of this interaction for receptor endocytosis by demonstrating that Barbadin blunts clathrin-mediated endocytosis of three prototypical GPCRs (β2AR, V2R and AT1R) without interfering with the translocation of β-arrestin to the receptor, nor with the interaction of AP2 with other components of the endocytic machinery. When used to dissect the role of the β-arrestin/AP2 interaction in GPCR signalling, Barbadin was found to inhibit the activation of ERK and cAMP production elicited by receptor stimulation, demonstrating that the β-arrestin/AP2 complex plays a role not only in receptor trafficking but also in signalling.

This work also illustrates that virtual screening can successfully identify small molecule inhibitors of protein–protein interactions. Despite the fact that identifying protein–protein interface inhibitors remains challenging due to the large surfaces of these interfaces and their planar architecture, a number of successes have now been reported for protein complexes bearing favourable quaternary architectures^41^,^42^. In this context, the fact that the groove delineating the β-arrestin binding site on the β2-adaptin ear platform subdomain is well-defined^19^,^20^ undoubtedly contributed to conduct reliable docking calculations that led to the identification of a validated inhibitor.

The docking pose and thermal-shift assay experiments suggest that Barbadin binds directly to the platform domain of β2-adaptin and relies on residues that are also involved in the interaction with β-arrestins. Indeed, the role of the benzyl-phenyl moiety of Barbadin in mimicking Phe388 and Phe391 of β-arrestin for binding β2-adaptin, is supported by the observations that analogues lacking one of the two phenyl rings (analogues A, B and compound 43) or altering the distance and flexibility between these rings (analogue C) showed lower inhibitory action on the β-arrestin/β2-adaptin interaction. Consistent with such a mode of binding that competes for the binding of β-arrestin C-tail to the platform domain of β2-adaptin, Barbadin inhibited the interaction between β2-adaptin and β-arrestin in a reversible manner without affecting the recruitment of β-arrestin to the receptor. Such selectivity may not be surprising given that the targeted domain of interaction between AP2 and β-arrestin is remote from the polar core of β-arrestin that interacts with the receptors^16^,^43^. Furthermore, as the engagement of AP2 by β-arrestin follows the recruitment of β-arrestin to the receptors^24^, blocking AP2 binding would not be predicted to influence the initial β-arrestin binding to the receptor. The topologically driven selectivity of Barbadin is also illustrated by its lack of effect on the interaction between β2-adaptin and other components of the CCPs such as clathrin and epsin, which bind distinct sites on β2-adaptin compared with β-arrestin. Consistent with the selectivity of Barbadin, the formation of CCPs is not disturbed as demonstrated by co-localization of AP2 and clathrin.

**Figure 8:**
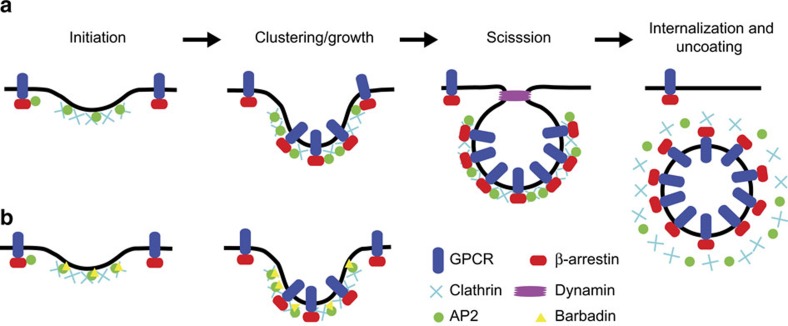
Model for the biogenesis of clathrin-coated pits (CCPs) and Barbadin's effect on GPCRs endocytosis. (**a**) Initiation of CCPs formation involves the association of AP2 with phosphatidylinositol 4,5-bisphosphate (PtdIns(4,5)P2) at the plasma membrane (PM) and the recruitment of clathrin to AP2 at sites of nucleation. Growth and stabilization of coated-vesicles requires the recruitment of additional AP2 and clathrin, and endocytic accessory proteins like epsin (not shown here) and β-arrestins. Agonist-activated GPCR/β-arrestin complexes accumulate in CCPs through β-arrestin's interactions with AP2 (β2 and μ subunits) and clathrin, stabilizing further the growing coated-vesicles. Stabilization of GPCR/β-arrestin complexes and the recruitment of endocytic effectors like dynamin allows the matured coated-vesicles to commit for invagination and scission from the PM, which is followed by the uncoating (that is, release of AP2 and clathrin) of internalizing vesicles. (**b**) In the presence of Barbadin, initiation of CCPs still occurs because clathrin and AP2 are effectively recruited at sites of nucleation. Some receptor/β-arrestin complexes will still coalesce into nucleating CCPs through β-arrestin's binding with clathrin and its low affinity interaction to AP2 (for example, via the μ subunit). Because Barbadin prevents the interactions of β-arrestin with the appendage domain on the β2-subunit of AP2, which prevents the stable formation of sufficient GPCR/β-arrestin complexes in CCPs, the maturation of the coated-vesicles is hampered, and receptor internalization impeded.

As predicted by its inhibitory action on the β-arrestin/β2-adaptin interaction, Barbadin significantly inhibited receptor endocytosis as quantified by flow-cytometry and BRET, and imaged by fluorescence microscopy experiments monitoring V2R and β-arrestin cell surface localization. Although Barbadin did not affect the recruitment of β-arrestin to the plasma membrane upon V2R stimulation, it prevented its subsequent trafficking to endosomes. This inhibition resulted in a large fraction of the β-arrestin accumulating at the cell surface and co-localizing with AP2 and clathrin in CCPs. This finding suggests that the β-arrestin-dependent accumulation of V2R-cargo into forming CCPs is a multi-step process involving numerous interactions with structural and endocytic accessory proteins of the coat (Fig. 8). Interestingly, it also suggests that β-arrestin's interaction with β2-adaptin is essential in the maturation of clathrin-coated vesicle-mediated internalization (for example, accumulation of the V2R/β-arrestin complex into clathrin-coated vesicles at plasma membrane and their final invagination). In that respect, the effect of Barbadin is reminiscent of the expression of an AP2 complex lacking the appendage domain of α-adaptin—which like the β-appendage that binds β-arrestin for initiating GPCR endocytosis, also recruits other regulatory/accessory proteins on its platform domain for the internalization of other classes of receptors—where it prevented the maturing and invagination of CCPs^13^,^44^. The fact that Barbadin did not prevent the clustering of receptor/β-arrestin complexes in CCPs is not unexpected (Fig. 6a–c), considering that β-arrestin also forms low affinity interactions with clathrin through its carboxy tail^45^, and with AP2 via its μ2 subunit through β-arrestin's N-terminal domain^46^. Indeed, when preventing the former interaction, the inhibitory effect of Barbadin for clustering receptor/β-arrestin complexes in CCPs was more readily observed. The robust Barbadin-promoted inhibition of both agonist-promoted BRET between β2-adaptin-YFP and β-arrestin-RlucII, as well as endocytosis for the β2AR, V2R and AT1R support previous observations demonstrating that the interaction between the carboxyl tail of β-arrestin and the β2-adaptin subunit of AP2 is preponderant in the initial steps of agonist-promoted internalization of many GPCRs^17^. The selective action of Barbadin on endocytosis processes mediated by the β-arrestin/AP2 complex was supported by its lack of effect on the ligand-independent and ligand-promoted internalization of transferrin and endothelin-1 receptors, respectively, which are β-arrestin- and AP2-independent^24^,^31^,^32^. The demonstration that Barbadin blocks receptor endocytosis, not only in engineered cells heterologously expressing GPCRs but also in vascular smooth cells endogenously expressing AT1R, paves the way for its use to assess the role of the β-arrestin/β2-adaptin complexes in native systems.

The observed effects of Barbadin on the ERK1/2 and cAMP signalling activities of the receptors confirm the role played by endocytosis in the control of specific GPCR signalling modalities^9^,^11^,^12^ and more particularly unambiguously demonstrate the essential, but perhaps underappreciated, role of the β-arrestin/AP2 interaction in this process. Barbadin completely blocks the V2R-stimulated ERK1/2 activation confirming the role of β-arrestin in this signalling pathway for V2R (ref. 38), but the result also points to a central role of endocytosis- and CCPs-mediated activation of MAPK by the receptor/β-arrestin complex. This finding is consistent with the emerging role of β-arrestin in CCPs for MAPK signalling, which was described for the Bradykinin B2 receptor and recently shown for the β1-adrenergic receptor^47^,^48^. Thus, inhibition of β-arrestin signalling in both CCPs and endosomes (because Barbadin prevented the trafficking of receptor/β-arrestin complexes to these intracellular compartments) most likely contributes to the total blockade of V2R-mediated activation of ERK1/2 by Barbadin. This inhibitory action of Barbadin on ERK1/2 activation cannot be attributed to a non-specific off-target effect of the compound, on other components of the MAPK pathway since it did not affect ERK1/2 activation promoted by the EGF receptor. These results are therefore consistent with the notion that spatio-temporal GPCR-stimulated ERK1/2 activation occurs following clustering of receptor/β-arrestin in CCPs and endocytosis of the complex in endosomes^9^,^49^.

The effect of Barbadin on the cAMP production stimulated by both β2AR and V2R also provides new insights on the spatio-temporal regulation of signalling as it brings strong support to the emerging notion that GPCR-stimulated cAMP production continues after endocytosis of Gs-coupled receptors^13^,^50^,^51^,^52^,^53^,^54^,^55^,^56^, contrasting with the classical view that receptor endocytosis contributes only to the termination of cAMP signalling. Sustained interaction between receptor and β-arrestin at the plasma membrane upon inhibition of endocytosis could also directly contribute to the reduced cAMP accumulation by preventing reactivation of the receptor at the plasma membrane. The effect was specific to receptor-mediated cAMP production and did not result from an off-target effect on Gs or the adenylyl cyclase since Barbadin had no effect on the Gs activity as directly monitored by BRET or cAMP generation resulting from a direct activation of adenylyl cyclase by forskolin. The extension of endocytosis-dependent cAMP production to the V2R is particularly interesting given the fact that this receptor does not recycle to the plasma membrane following endocytosis but is instead retained for an extended period of time in endosomal compartments before being targeted for degradation^57^. This result excludes the possibility that the diminution of V2R-promoted cAMP production following Barbadin treatment could result from the accumulation of desensitized receptor at the plasma membrane that cannot be replaced by re-sensitized ones upon inhibition of endocytosis.

Barbadin is distinct from the existing endocytosis blockers as it selectively interferes with the β2-adaptin/β-arrestin interaction and thus solely blocks endocytic processes that are dependent on the interaction between β-arrestin and the appendage domain of the β-subunit of AP2. Existing pharmacological inhibitors such as dynasore^58^, dynoles^59^ or dyngos^30^ inhibit all dynamin-dependent endocytic pathways, while pitstop1/2 (ref. 28) inhibit both clathrin-dependent and -independent endocytosis^29^. Although we cannot rule out the possibility that Barbadin interacts with other targets in cells, we found that it did not directly act on other components of the clathrin endocytic machinery, nor on important signalling effectors (for example, β-arrestin- and G protein-dependent signalling promoted by GPCRs, cAMP production by adenyl cyclase and MAPK activation promoted by other receptors like EGFR) supporting a selective mode of action. Barbadin's selectivity of action on β-arrestin/AP2 interaction allows the dissection of the relative contribution of this complex to the regulation of GPCRs endocytosis and signalling, independently of other processes controlling cell surface density, and thus represents a valuable addition to the existing pharmacological toolbox^60^.

## Methods

### Materials

DMEM, FBS, penicillin, streptomycin, PBS and G418 were from Wisent Inc. Cell culture plates and dishes were purchased from BD Biosciences. Arginine vasopressin (AVP), isoproterenol (ISO), Angiotensin-II (AngII), Epidermal Growth Factor (EGF), endothelin 1 (ET1), sigmacote, poly-L-ornithine hydrobromide, 3-isobutyl-1-methylxanthine (IBMX), Triton X-100, mouse monoclonal anti-β_1_- and β_2_-adaptins (AP1/2, clone 100/1, #A4450), rabbit Anti-Flag antibody (#F7425), anti-Flag (M2, #F3165) and anti-mouse or anti-rabbit HRP conjugated antibodies (#A9044 and #A9169, respectively) were from Sigma-Aldrich. Poly-d-lysine was obtained from MatTek Corporation. Linear polyethylenimine 25-kDa (PEI) was from Polysciences. BARR3978 antibody against β-arrestin was previously described^61^. Phospho-p42/p44 (pERK1/2, #9106) and p42/p44 (ERK1/2, #9102) antibodies were from Cell Signaling Technology. Antibodies against clathrin heavy chain (Clone 23, 610499) and adaptin β (Clone 74, #610381) were obtained from BD Biosciences. Epsin1/2 rabbit antibody was kindly provided by Peter McPherson (McGill University). Dithiobis succinimidyl propionate (DSP) was from Pierce. Coelenterazine H, coelenterazine 400 A and Prolume Purple were from Nanolight Technology. Mouse monoclonal anti-HA antibody (HA.11) was purchased from Covance (#MMS-101 P). Anti-mouse Alexa Fluor 647 secondary antibody (A-21463), fluorescent transferrin conjugate (Alexa Fluor 633, T23362), puromycin and salmon sperm DNA were bought from Invitrogen. Pitstop2 was from Abcam (ab120687) and Dyngo (S7163) from SelleckChem. cAMP dynamic-2 kit was from Cisbio. Costar v-bottom polypropylene 96-wells plates were from Corning. White Optiplate 96-wells microplates and 384-well Proxiplates were purchased from PerkinElmer. Lumitrac 200 384-wells plates were from Greiner Bio-one. X-treme GENE HP reagent was from Roche and Strep-Tactin Sepharose beads from IBA Gmbh. β-arrestin1 C-terminal peptide (sequence: DDDIVFEDFARQRLKGMKDD) was synthesized by Genscript, while CPM dye was purchased from ThermoFisher Scientific. Protein concentration were determined using a detergent compatible protein assay kit from Bio-Rad.

Differential scanning fluorimetry was done in a RotorGeneQ from Qiagen. Luminescence and BRET reading were performed in the Mithras LB 940 multidetector plate reader from Berthold Technologies. Cell surface expression of receptors was analysed in a LSR II flow cytometer from BD Biosciences. Radioactivity was counted using a PerkinElmer Wizard 1470 automatic γ-counter. For microscopy, colocalization analyses were performed using Zen software from Zeiss. Time-resolved fluorescence resonance energy transfer was read in an Artemis plate reader from Cosmo Bio USA.

### Virtual screening

Virtual screening by docking has been performed on a computer cluster of 10 Intel Core 2 Quad 2.6 GHz processors. The SD file of the Myriascreen compounds collection (library number T990000) has been requested to Sigma-Aldrich (www.sigmaaldrich.com). Canonicalization and three-dimensional coordinates generation of the compounds were performed using Chemaxon Standardizer, JChem 5.1.2 (www.chemaxon.com). Starting from the β2-adaptin/β-arrestin co-crystal structure (PDB entry 2IV8), the AutoDockTools^62^ interface was used to prepare the binding site of β2-adaptin which was defined as a 30-Å cubic box centred on the β-arrestin helical peptide bound to the β2-adaptin ear platform subdomain. Docking was carried out using Autodock 4.0 (ref. 62) with 30 independent runs of simulation per compound docked. Autodock scoring function was used to rank compounds by lowest energy. For visual inspection of the best energy candidates, we chose to select any compounds within a range of 2.1 kcal mol^−1^ (s.e. of Autodock) from the best compound. The best 196 selected compounds, and the docked Barbadin analogues were visually analysed using PyMOL (PyMOL Molecular Graphics System, Version 1.4 Schrödinger, LLC) and the MOE package (Molecular Operating Environment (MOE), 2012.10, Chemical Computing Group Inc., 1010 Sherbooke St. West, Suite #910, Montreal, QC, Canada, H3A 2R7, 2012).

### Compounds acquisition and technical consideration

The 52 small organic compounds from the original screen were purchased from Sigma. Because Barbadin was later discontinued by Sigma, it was then acquired from Otava (#0129700278). Barbadin analogues were acquired from Enamine (see Supplementary Table 1 for catalogue numbers). All compounds were solubilized in 100% DMSO at a 10 mM stock concentration. To circumvent the possibility that Barbadin adheres to plastic, all assays were carried out using tips, tubes and 96-wells plates coated with Sigmacote, then rinsed extensively with water.

### Plasmids and constructs

Plasmids encoding myc-V2R (ref. 63), V2R-YFP (ref. 63), HA-ETAR (ref. 64), HA-AT1R (ref. 65), β-arrestin1-RlucII (ref. 65), β-arrestin2-RlucII (ref. 66), β-arrestin2-YFP (ref. 67), β-arrestin2-mCherry (ref. 65), β2-adaptin-YFP (ref. 68), GST- β2-adaptin(592–937) (ref. 19), flag-β-arrestin1 (ref. 6), β2AR-RLucII (ref. 26), V2R-RLucII (ref. 26), AT1R-RLucII (ref. 26), rGFP-CAAX (ref. 26) and rGFP-FYVE (ref. 26) were previously described. β2-adaptin-mCherry was generated by replacing the YFP moiety of β2-adaptin-YFP with that of the mCherry using the AgeI and BsrGI sites. β2-adaptin-CFP was generated by replacing the YFP moiety of β2-adaptin-YFP with that of the CFP from the pECFP vector. Clathrin light chain-YFP was purchased from addgene (Cambridge, MA). The LIEF/AAEA substitution in human β-arrestin2-YFP (β-arr2-ΔClath-YFP) was generated by complementation PCR reaction, using the forward primer 5- GGACACCAACGCAGCTGAAGCCGATACCAACTATGCC-3 with the reverse 5- TGGCGACCGGTGGATCCCGGCAGAGTTGATCATCATAGTCG-3 primer containing the BamHI site, and the reversed primer 5- TCGGCTTCAGCTGCGTTGGTGTCCACAGGGACATC-3 and forward primer 5-AGATCTCGAGCTCAAGCTTATGGGGGAGAAAC-3 containing the HindIII site. The PCR product from the amplified complementing fragments was cloned into BamHI/HindIII sites by Gibson assembly (NEB). The β2AR construct was generated by inserting a polymerase chain reaction (PCR)–amplified fragment encoding human β2AR into a pcDNA3.1 vector, between HindIII/XbaI. The β2-adaptin(700–937)-StrepTagII construct was generated by inserting a PCR-amplified fragment encoding human β2-adaptin (residues from 700 to 937) into a pRSETA vector (Invitrogen), between the BamHI and Acc65I restriction sites. To generate HA-β2AR-Venus encoding plasmid, the Venus sequence was first PCR-amplified and introduced into the previously described pIRESP-HA vector^69^. Then, the β2AR sequence was PCR-amplified and inserted into pIRESP-HA-Venus vector by recombination using In-Fusion PCR Cloning Kit from Clontech (Mountain View, CA). The HA-β2AR-Venus stable cell line was generated by transfecting pIRESP-HA-β2AR-Venus in HEK293T followed by polyclonal selection of expressing cells using 3 μg ml^−1^ of puromycin. The expression vectors pIREShygro3 and pIRESpuro3 were from Clontech (Mountain View, CA). The plasmid encoding HA-V2R-Venus was made in two cloning steps. First, HA-V2R gene was PCR-amplified from pcDNA3.1(+)3HA-V2R plasmid from Missouri S&T cDNA resource centre (Rolla, MO), keeping one HA tag and removing the stop codon. The PCR product was then inserted in pIRESpuro3a vector. Then, the Venus sequence was PCR-amplified and inserted in pIRESpuro3a-HA-V2R. The resulting HA-V2R-Venus fusion sequence was then inserted into pIREShygro3 vector, yielding pIREShygro3-HA-V2R-Venus plasmid. The HA-V2R-Venus stable cell line was generated by transfecting pIREShygro3-HA-V2R-Venus in HEK293T followed by polyclonal selection of expressing cells using 100 μg ml^−1^ of hygromycin B. All constructs were verified by DNA sequencing.

β2-adaptin deletion in HEK293 cells (CRISPR-β2Ad) were generated using the CRISPR/Cas9 system PspCas9(BB)-2A-Puro (Px459v2) vector (Gift from Feng Zhang: Addgene plasmid #62988)^70^ and the gRNA target sequences (5′-GTATTTCACAACCAATAAAAA-3′ and 5′-GGTCTGGCTACCTGCAGTAA-3′) against the human AP2B1 gene (GenScript gRNA database). Each gRNA was cloned into the Px459v2 vector, and both constructs were transfected in HEK293 cells using Lipofectamine 2000 according to the manufacturer's instructions. The next day, medium supplemented with puromycin (2 μg ml^−1^) was added to cells for 48 h and cells were serially diluted and plated in 96-wells plates for generating individual colonies. Colonies were then verified for β2-adaptin expression by western blot and by PCR genotyping using primers flanking the deleted region. Positive clone #5 (hereafter named CRISPR-β2Ad-LY5 cells) was selected.

### Cell culture

HEK293T is the cell line in which BRET-based biosensors have been developed in Dr Bouvier's laboratory and this cell line was used for all the BRET and FACS experiments. HEK293SL are a subclone derived from regular HEK293 cells (Ad5 transformed) and were selected in Dr Laporte's laboratory. These cells have a cobblestone appearance and a better adherence as compared with regular HEK293T cells, making them more amenable to microscopy. HeLa is a cell line expressing a higher level of native TfR, compared to HEK293T cells, and were consequently used for monitoring TfR expression by FACS. HEK293T, HEK293SL and HeLa cells were cultured in DMEM supplemented with 10% FBS, 100 units per ml penicillin and streptomycin and incubated at 37 °C in 5% CO_2_.

Primary rat aortic VSMCs were a gift from Dr. Marc Servant (Université de Montreal) and were used for endogenous AT1R internalization assay. These cells were grown in DMEM/high glucose supplemented with sodium pyruvate, 10% FBS, and 20 μg ml^−1^ gentamycin.

All cells were regularly tested for mycoplasma contamination (PCR Mycoplasma Detection kit, abm, BC, Canada).

### Bioluminescence resonance energy transfer (BRET) assays

HEK293T cells stably expressing β2-adaptin-YFP (ref. 24) were cultured as aforementioned with the addition of G418 in the supplemented DMEM. Forty-eight hours before the experiments, 10 μg of total DNA (adjusted with salmon sperm DNA) was used to transfect 6 × 10^6^ cells per ml in 10-cm dishes using 25-kDa linear PEI as a transfecting agent (3:1 PEI/DNA ratio). Cells stably expressing β2-adaptin-YFP were transfected with either 250 ng of V2R, or 2.5 μg of β2AR, or 3.0 μg of AT1R and with either 100 ng of β-arrestin1-RlucII or β-arrestin2-RlucII for the compounds screen, the concentration-response curves, the one-point stimulation experiments, as well as for the kinetics that monitors the interaction between β2-adaptin and β-arrestin. To monitor β-arrestin recruitment to either V2R or β2AR, HEK293T cells were transfected with 5 μg of V2R-YFP or 3 μg of β2AR-YFP and 500 ng of β-arrestin1-RLucII or 120 ng of β-arrestin2-RLucII. For the pre-screen experiment assessing compounds interference with BRET signals, HEK293T cells were transfected with 100 ng of β-arrestin1-RlucII. On the day of the BRET experiment, cells were washed, detached and resuspended in Tyrode's buffer. Cells (10^5^ cells per well) were distributed in 96-wells microplates. DMSO or compounds selected from the virtual screening were preincubated for 30 min at indicated concentrations, always keeping DMSO at 1% final concentration in each well. Cells were treated with AVP or ISO at the indicated times and concentrations, at room temperature. Coelenterazine H (for YFP BRET acceptor) or coelenterazine 400 A (for GFP10 BRET acceptor) was added at a final concentration of 2.5 μM five minutes before measurements. Readings were collected with a Mithras LB 940 multidetector plate reader (Berthold Technologies) to integrate signals detected in the 485/20 nm and 530/25 nm windows for the Renilla luciferase (RlucII) and YFP light emissions, respectively, and in the 400/70 nm and 515/25 nm windows for the RlucII and GFP10 light emissions, respectively. The BRET signal was determined by calculating the ratio of the light intensity emitted by the YFP or GFP10 over the light intensity emitted by the RlucII. Net BRET values were obtained by subtracting the background BRET signals detected when RlucII was expressed alone. Ligand-promoted BRET was calculated by subtracting the BRET ratio obtained in the absence of agonist from the BRET ratio detected in its presence.

### BRET imaging

HEK293SL cells seeded on poly-d-lysine-coated glass-bottom 35-mm culture dishes were transfected with 100 to 200 ng per dish of AT1R-RlucII or V2R-RlucII, respectively, with either 400 ng per dish of rGFP-CAAX or 300 ng per dish of rGFP-FYVE, using X-treme GENE HP reagent (Roche). Two days post transfection, cells were washed once with 1 ml of modified Hank's balanced salt solution (138 mM NaCl, 5.33 mM KCl, 0.44 mM KH_2_PO4, 0.33 mM Na_2_HPO_4_, 4.16 mM NaHCO_3_, 1.0 mM CaCl_2_, 1.0 mM MgCl_2_, 10 mM HEPES, pH 7.4) and set on the microscope. BRET images were obtained using Nikon Ti-U microscope equipped with × 60 objective (Apochromat TIRF, NA 1.49, Nikon) and EMCCD camera (EM N2 1024, Nüvü Cameras) with filter changer (Lambda 10-2, Sutter instrument). Prolume Purple (final concentration of 10 μM) was added and images were obtained using photon counting feature of the camera. The exposure time of each image was 200 msec. Images were obtained with or without a filter corresponding to the BRET acceptor (480LP) wavelength. The filter was switched after taking every 50 images, and the final image was obtained by integrating successive 500 images applying the same filter to the camera. BRET ratio images were generated using pixel arithmetic functions of MetaMorph software version 7.8 (Molecular Devices) as follows: Pixel hue: BRET level calculated by dividing the counts of acceptor luminescence images with total luminescence images, and allocated to default rainbow hue (lowest in purple and highest in red); pixel brightness: the value of donor images with auto brightness.

### Protein purification

Plasmid for β2-adaptin(700–937)-StrepTagII was transformed in E. coli BL21 pLysS cells that were then grown at 37 °C in TB medium containing 100 μg ml^−1^ ampicillin and 50 μg ml^−1^ chloramphenicol to an absorbance at λ=600 nm (A600) of 0.6. Protein expression was induced with 0.2 mM IPTG and cells were grown overnight at 30 °C. Pelleted cells were resuspended in lysis buffer (100 mM Tris–HCl, pH 8.0, 150 mM NaCl, 1 mg ml^−1^ lysozyme, 5% glycerol, PMSF 0.5 mM, TCEP 50 μM and 1 mM EDTA) and frozen at –80 °C. For purification, cells were thawed and lysed by mild sonication. After cell debris removal by centrifugation, lysates were incubated with rinsed Strep-Tactin Sepharose beads (IBA) at 4 °C for one hour on a rotator. The protein was subsequently eluted and purified further by passage over a sepharose column (Bio-Rad) and a gel filtration column. Protein was snap frozen in liquid nitrogen and stored at −80 °C for long-term storage.

### Differential scanning fluorimetry assay

The thermal denaturation of β2-adaptin (residues 700–937, molecular weight 26.88 kDa) was monitored by CPM (7-Diethylamino-3-(4′-Maleimidylphenyl)-4-Methylcoumarin) dye fluorescence changes upon conjugation to the cysteines of β2-adaptin that become exposed upon unfolding. The amount of 2.1 μg purified β2-adaptin was diluted in 100 μl final volume using phosphate buffered saline (PBS) to the concentration of 0.78 μM. Samples containing 0.5 mM Triton X-100 were incubated for 30 min without Barbadin (DMSO), with 10 μM, 33 μM or 100 μM Barbadin, or with 20 μM β-arrestin1 C-terminal peptide (sequence: DDDIVFEDFARQRLKGMKDD), synthesized by Genscript, USA) before the addition of CPM dye (ThermoFisher Scientific, Switzerland). CPM dye of 3 mg ml^−1^ in DMSO was diluted 1:80 in PBS and 10 μl of the dilution was added to each sample to a final concentration of 9.3 μM. Each sample was split into three 25 μl aliquots. The samples were then heated from 30 °C to 90 °C at 6 °C min^−1^ in a RotorGeneQ and the fluorescence of the CPM dye was monitored using 365 nm excitation and 460 nm emission. RotorGeneQ Series Software was used for data analysis. The first derivative of the fluorescence data was used to determine the melting temperatures.

### Flow-cytometry for assessing receptor cell surface expression

For monitoring V2R and β2AR cell surface expression, HEK293T cells stably expressing either HA-V2R-Venus or HA-β2AR-Venus were seeded in 150mm dishes. For monitoring ET_A_R cell surface expression, HEK293T cells were transfected with HA-ET_A_R in 100 mm dishes, using the PEI procedure previously described in the methods. Forty-eight hours later, cells were harvested, rinsed with PBS, then detached and resuspended in Tyrode's buffer. Cells (1 × 10^6^ cells per well) were distributed in v-bottom 96-well plates. DMSO, Barbadin (100 μM), Pitstop2 (100 μM) and Dyngo-3a (30 μM) were preincubated for 30 min (final 1% DMSO content in each well). Cells were treated at room temperature, in the absence or presence of AVP (100 nM), ISO (10 μM) or ET1 (10 nM) for the indicated times, then put on ice for the rest of the experiment. Plates were quickly spun at 4 °C and supernatant from each well was removed using a vacuum line. Cells were resuspended in Tyrode supplemented with 1% BSA (Tyrode/BSA) containing mouse monoclonal anti-HA antibody (1:1,000) to label cell surface receptors. After 20 min incubation, plates were quickly spun then resuspended in Tyrode/BSA containing anti-mouse Alexa Fluor 647 secondary antibody (1:1,000). After 30 min incubation and protected from light, plates were quickly spun, and cells washed and resuspended in Tyrode and kept on ice. Cells were analysed through a LSR II flow cytometer set to detect YFP and Alexa Fluor 647 nm in distinct channels (Supplementary Fig. 8). For monitoring transferrin receptor surface expression, HeLa cells were seeded in a 150 mm dish and incubated for 48 h before being processed. One hour before the experiment, cells were serum-starved, then rinsed with PBS and resuspended in Tyrode's buffer. Cells were distributed in centrifuge tubes (500 μl of resuspension per tube with 1 × 10^6^ cells each). DMSO, Barbadin (100 μM) or Pitstop2 (100 μM) were preincubated for 30 min at room temperature, then cells were put on ice for 10 min. Cells were incubated with Alexa Fluor 633-conjugated transferrin (100 μg ml^−1^) for 30 min at 4 °C in the dark, rinsed with cold PBS and shifted to 37 °C for 15 min, except for a control tube that was kept on ice. Cells were then pelleted, washed, acid-washed (0.1 M glycine, 150 mM NaCl, pH 3), resuspended in Tyrode containing 1% BSA and analysed in a LSR II flow cytometer (Alexa Fluor 647 channel).

### VSMC internalization assay

[^125^I]-AngII was prepared with the Iodogen method, and specific activity was determined from self-displacement and saturation experiments, as previously described^65^. Receptor internalization was performed as described previously^26^. Briefly, VSMCs were plated at a density of ∼7 × 10^4^ cells per well in poly-L-ornithine-coated 24-well plates. When cells were confluent (24–48 h after plating), they were starved in DMEM with 20 mM HEPES (DMEM-H) for 2 h before Barbadin treatment. Cells were incubated in either DMSO or Barbadin (20 μM) for 30 min at 37 °C, before adding [^125^I]-AngII (0.2 nM in 0.1% BSA binding buffer) for 15 min at 37 °C. Binding and internalization of [^125^I]-AngII was terminated by rapidly rinsing the cells twice with ice-cold PBS and then either rinsing once with ice-cold PBS to obtain total binding, or with 50 mM acetic acid in 150 mM NaCl (pH 2.7) for 10 min to determine the internalized receptors (acid-resistant). Cells were solubilized in 0.5 M NaOH, 0.05% SDS and radioactivity was counted using a Wizard 1470 automatic γ-counter. Per cent receptor internalization was calculated from the ratio of acid-resistant binding over total binding. Non-specific binding was determined in the presence of 1 μM unlabelled AngII.

### Fluorescence microscopy

For β-arrestin colocalization with β2-adaptin experiments, HEK293T stably expressing human V2R cells were seeded in 35 mm optical glass-bottom culture dishes with poly-L-lysine. Twenty-four hours later, cells were transfected with 300 ng of β-arrestin2-mCherry, 400 ng of β2-adaptin-YFP with pcDNA3.1 for up to 12 μg of total DNA using conventional calcium phosphate co-precipitation method. For colocalization of β-arrestin2 with β2-adaptin and clathrin, CRISPR-β2Ad-LY5 cells were seeded on coverslips coated with poly-Ornithine and transfected with 2 μg of HA-V2R, 400 ng of β2-adaptin-CFP, 300 ng of β-arrestin2-mCherry, 150 ng of clathrin-YFP and pcDNA3.1 for up to 12 μg of total DNA using lipofectamine 2000 according to the manufacturer's instructions. Media were changed the next day and experiment performed 48 h post transfection. Cells were serum-starved for 30 min, then pretreated with 10 μM Barbadin or vehicle (DMSO) for an additional 30 min before stimulation with 1 μM AVP for 2.5 min. Before mounting coverslips on slides, cells were first washed in PBS, then incubated for 10 min in PBS-containing 4% Paraformaldehyde (PFA, v/v). Images of live cells were collected on a Zeiss LSM-510 Meta laser scanning microscope with a × 63/1.4 oil objective lens using excitation/emission filter sets: 543 nm/560 nm (Long Pass) for mRFP, and 514 nm/530–600 nm (Band Pass) for YFP. Images of fixed cells were collected on a Zeiss LSM-780-NLO laser scanning microscope with a × 63/1.4 oil DIC M27 objective lens using 543 nm, 514 nm and 458 nm for mCherry, YFP and CFP excitation, and a GaAsP spectrum detector set at 588–695 nm, 517–580 nm and 464–517 nm, respectively. Co-localization analysis was performed using regions of interest (ROI) and analysed using Zen software.

### GST-pulldown assays

Plasmid DNA for GST and GST-β2-adaptin(592–937) were transformed in *E. coli* BL21 cells. Overnight cultures were grown in super-broth medium supplemented with ampicillin (100 μg ml^−1^), diluted to an *A*_600_ of 0.4 in the same medium and grown for another 1 h 30 at 37 °C to reach an *A*_600_ of 0.8 (log phase). Cultured cells were then induced with 0.1 mM isopropyl-1-thio-β-D-galactopyranoside (IPTG) for 5 h at 30°C. Cells were then pelleted, washed once with PBS and resuspended in PBS containing 1 mM phenylmethylsulfonyl fluoride (PMSF), 2 mg ml^−1^ lysozyme and incubated for 15 min on ice. Cells were lysed by immediately adding Triton X-100 (1% v/v) followed by two freeze-thaw cycles in liquid nitrogen. Solubilized cells were sonicated (3 × 15 s) on ice and centrifuged at 13,000 r.p.m. for 10 min. Glutathione-sepharose beads were added to the supernatant and gently shaken at 4 °C for 2 h. Beads were washed three times with cold PBS containing 1% Triton X-100, then three times with cold PBS. Protein concentration was determined using a detergent compatible protein assay kit, and the integrity of the fusion proteins was analysed by SDS-polyacrylamide gel electrophoresis and by Coomassie staining. To prepare cell lysate, HEK293T (1.2 × 10^6^) were plated in 100 mm dishes and transfected 24 h later with 5 μg of Flag-β-arrestin1 and pcDNA3.1 (to a total of 12 μg) using conventional calcium phosphate co-precipitation method. After overnight incubation, transfection medium was replaced with fresh MEM. Twenty-four hours later, cells were washed with PBS and lysed in a glycerol buffer containing 1 mM PMSF, 25 μg ml^−1^ leupeptin, 2.5 μg ml^−1^ aprotinin and 1 mM pepstatin. Cell lysates were cleared by centrifugation (14,000 r.p.m. for 15 min). Seven micrograms of GST or GST-β2-adaptin(592–937) were incubated for 1 h at RT with or without Barbadin (200 μM) in 100 μl PBS, then 100 μl of cell lysate was added and left 2 h at 4 °C on a Nutator. Beads were spun and washed five times in glycerol buffer and resuspended in SDS sample buffer (8% SDS, 25 mM Tris–HCl, pH 6.5, 10% glycerol, 5% 2-mercaptoethanol, 0.003% bromophenol blue). Proteins were resolved by electrophoresis on a 10% gel, transferred onto nitrocellulose and analysed by western blot using mouse monoclonal anti-Flag (1:1,000) and anti-clathrin (1:1,000) antibodies, or stained with Coomassie blue for GST protein detection.

### Co-immunoprecipitation

Co-immunoprecipitation experiments were performed as previously described^67^. Briefly, HEK293SL cells transfected with 3 μg V2R and 500 ng of Flag-β-arrestin2 were serum-starved for 30 min, and then pretreated for 20 min with DMSO or Barbadin (50 μM) before stimulation with AVP (1 μM) for 2.5 and 5.0 min. Stimulation was stopped by adding dithiobis succinimidyl propionate (DSP) to cells (2 mM). Cells were then washed in 50 mM Tris–HCl (pH 7.5) and lysed in RIPA buffer (50 mM Tri–HCl, pH 7.4, 1% NP-40 (v/v), 0.5% Na-Deoxycholate (w/v), 0.1% sodium dodecyl sulfate (w/v), 150 mM NaCl, 2 mM EDTA, 50 mM NaF). Total cell lysates (TCL) were immunoprecipitated with anti-AP1/2 antibody (25 μg) and then analysed by western blot using anti-adaptin (BD Biosciences, 1:1,000) for TCL and AP1/2 (1:1,000) for IP samples, anti-epsin (1:500) or anti-Flag antibodies (1:1,000), and secondary HRP antibodies (1:7,000)^61^. AP1/2 antibody has been reported to detect both the β1 and β2 subunits, which have different sizes. However, in HEK293SL, AP1/2 antibody mainly immunoprecipitated and recognized a protein of ∼105 kDa (Supplementary Fig. 9), which we validated to be immune-reactive for β2-adaptin using a specific antibody against this subunit (BD Bioscience). This was confirmed using β2-adaptin knock out cells (CRISPR-β2Ad-LY5, Supplementary Fig. 7).

### cAMP assay

Intracellular cAMP accumulation was measured using the cAMP dynamic-2 kit, a competitive immunoassay based on homogeneous time-resolved fluorescence technology. HEK293T cells, as well as HEK293T stably expressing either V2R or β2AR were starved for 4 h in DMEM before being processed. Cells were rinsed once in PBS and once in cAMP-assay buffer (PBS with 1% BSA and 0.5 mM IBMX), detached and resuspended in the same buffer, then seeded (4 × 10^4^ cells per well) in 96-well microplates. For concentration-response curves, cells were pretreated or not (DMSO) with Barbadin at the indicated concentrations for 30 min (1% DMSO final concentration in each well), then treated at room temperature with vehicle, AVP (100 nM), ISO (1 μM) or forskolin (10 μM) for 15 min and put at −80 °C for 1 h. For kinetics experiments, cells were pretreated or not (DMSO) with Barbadin (50 μM) for 30 min, then treated with vehicle, AVP (100 nM) or ISO (1 μM) for the indicated times, and put at −80 °C for 1 h. Once thawed, cells (4 × 10^3^ cells per well) were transferred in 384-well plates and incubated with cAMP labelled with the dye d2, and anti-cAMP antibody labelled with Cryptate according to the manufacturer's protocol. Reading of the homogeneous time-resolved fluorescence signal was performed on an Artemis plate reader.

### ERK1/2 activation assay

HEK293T cells (10^5^ cells per well) were seeded in a 6-well plate and transfected with a V2R encoding plasmid. Forty-eight hours post transfection, cells were serum-starved for 30 min in MEM containing 20 mM HEPES, treated with either DMSO or Barbadin (50 μM) for 10 min, and then stimulated with vehicle (MEM), AVP (1 μM) or epidermal growth factor (EGF, 100 ng ml^−1^) for the indicated times. Treatment was stopped on ice with cold PBS, and cells were solubilized in × 2 laemmli buffer (250 mM Tris–HCl pH 6.8, 2% SDS (w/v), 10% glycerol (v/v), 0.01% bromophenol blue (w/v) and 5% β-mercaptoethanol (v/v)). Lysates were resolved on a 10% SDS–PAGE and analysed by western blot using anti-phospho-ERK1/2 (1:1,000) and anti-total-ERK1/2 (1:2,000). Signals from western blots were determined by densitometry analysis using the Image J software (NIH).

### Data and statistical analyses

Nonlinear regression analysis of the concentration-response and kinetics curves and statistical analyses (assuming similar variance between groups) were performed with GraphPad Prism software. For the concentration-response curves the ‘log inhibitor versus response–three parameters (Hill slope=−1)' fitting was used. Student's *t*-test or one-way analysis of variance (ANOVA) and Tuckey's post-hoc tests were performed as appropriate (see figure legends). Two-way ANOVA and Bonferroni's post-hoc tests (comparison between all groups) were used for statistical analysis of western blot signals for ERK and co-immunoprecipitation assays, as well as for compounds' effect on receptor endocytosis kinetics by FACS. Colocalization of signals from microscopy experiments was analysed using Pearson's correlation coefficient.

### Data availability

The authors declare that all data supporting the findings in this study are presented within the article and its Supplementary Information files and available from the corresponding author upon request. Amino acid sequences for hβ2-adaptin are provided in Supplementary Data 1.

## Additional information

**How to cite this article:** Beautrait, A *et al*. A new inhibitor of the β-arrestin/AP2 endocytic complex reveals interplay between GPCR internalization and signalling. *Nat. Commun.* **8**, 15054 doi: 10.1038/ncomms15054 (2017).

**Publisher's note:** Springer Nature remains neutral with regard to jurisdictional claims in published maps and institutional affiliations.

## Supplementary Material

Supplementary Information Supplementary Figures and Supplementary Table

Supplementary Dataset 1 Amino-acid sequences of human β2-adaptin
