# Supplementary material for: A new inhibitor of the β-arrestin/AP2 endocytic complex reveals interplay between GPCR internalization and signalling
Source: Nat Commun. 2017 Apr 18;8:15054. doi: 10.1038/ncomms15054 (PMC5399295; doi:10.1038/ncomms15054)
Supplement: Supplementary Information — Supplementary Figures and Supplementary Table [file ncomms15054-s1.pdf]

Sup. Figure S1 (Bouvier)

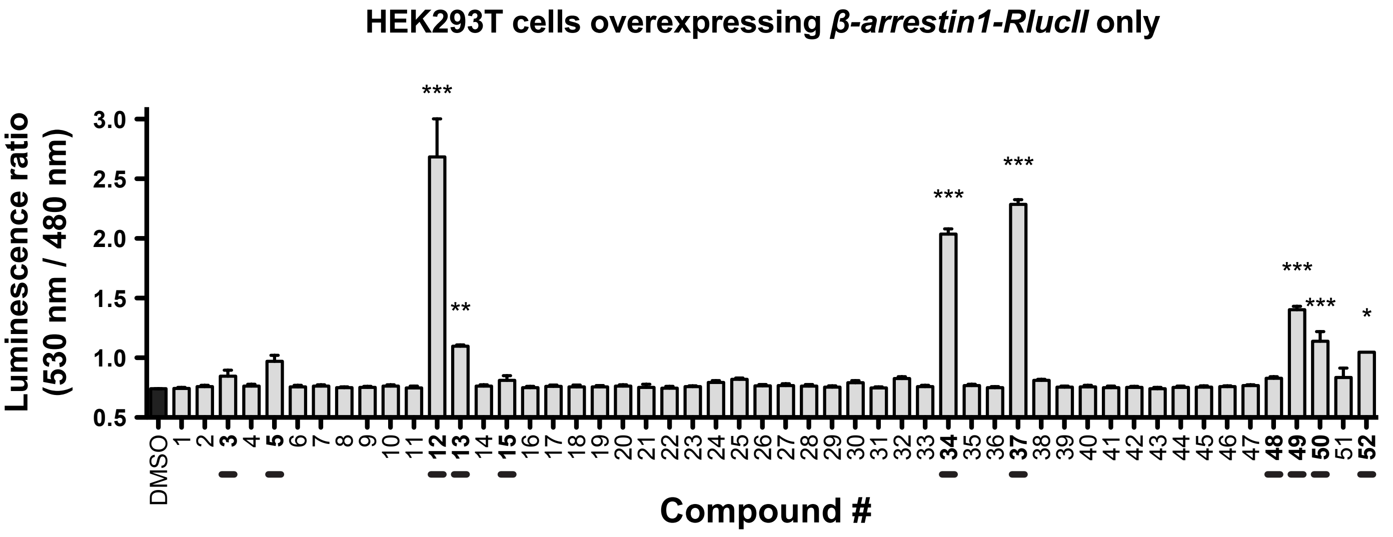

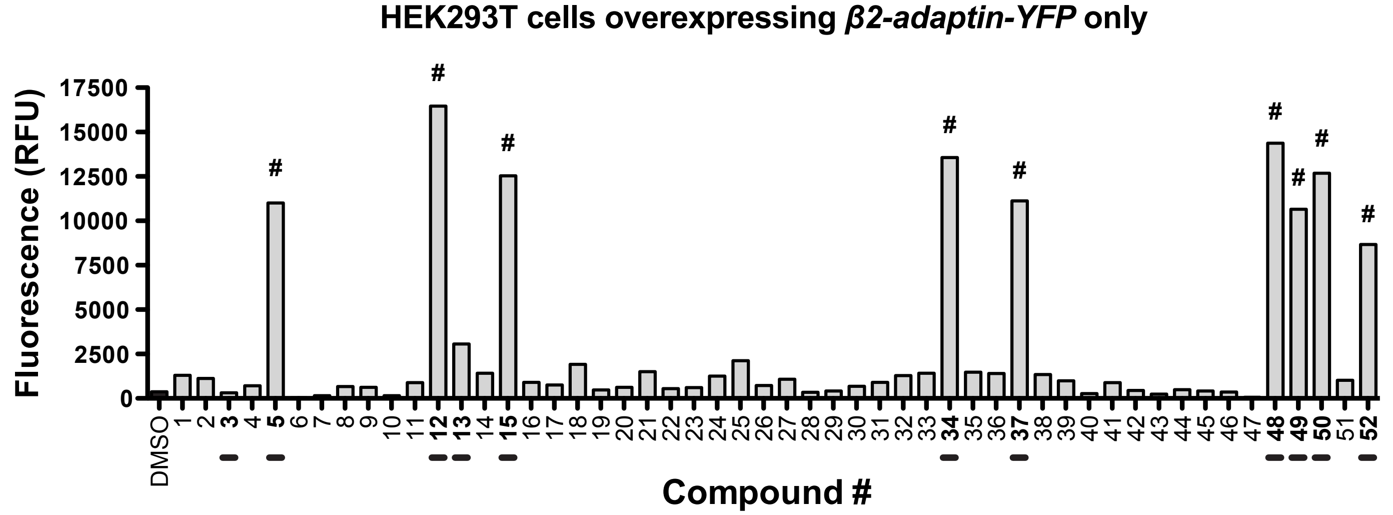

Supplementary Figure 1
Compounds effect on luminescence and fluorescence signals.

The 52 compounds selected for the screen have been tested at 100 µM in HEK293T cells overexpressing either the β-arrestin1-RLucII (**top graph**) or the β2-adaptin-YFP (**bottom graph**) construct alone. One-way ANOVA followed by Tuckey's post-hoc tests were performed with DMSO as control to assess statistical significance of the differences (*, $p<0.05$; **, $p<0.01$; ***, $p<0.001$) (**top graph**). Compounds showing signal higher than one standard deviation from the average were identified (#) (**bottom graph**). Eleven of the 52 compounds were coloured (underlined below the graphs). Of these, seven had a significant effect on the luminescence and 9 affected the fluorescence signal. They were discarded from further investigation.

Sup. Figure S2 (Bouvier)

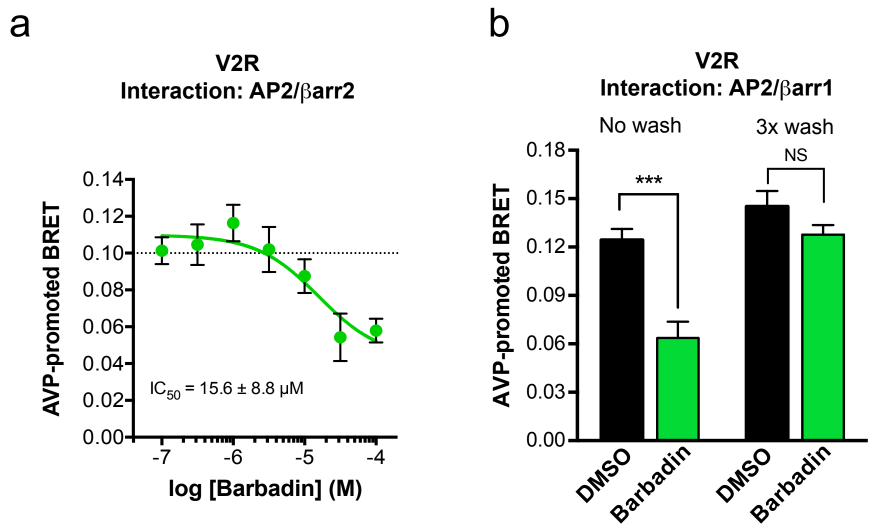

Supplementary Figure 2

Barbadin is a reversible inhibitor of the interaction between β2-adaptin and β-arrestin1/2.

(a) Concentration-response curves of Barbadin on V2R-induced (AVP, 100 nM) β2-adaptin/β-arrestin2 interaction assay (green line). Dotted line represents the level of AVP-promoted BRET upon pre-incubation with DMSO. Data are the mean ± S.E.M. of 3 independent experiments.

(b) BRET-based assay monitoring the AVP-induced interaction between β2-adaptin-YFP and β-arrestin1-RlucII. HEK 293T cells were pre-incubated with DMSO or Barbadin (100 μM) for 30 min prior to 45 min receptor stimulation with AVP (100 nM). When indicated, cells were washed 3-times with PBS for 10 min at 37°C before agonist stimulation. Data are the mean ± S.E.M. of 3 independent experiments and one-way ANOVA followed by Tuckey's post-hoc tests were used to assess statistical significance (NS, non-significant; ***, $p < 0.001$).

Sup. Figure S3 (Bouvier)

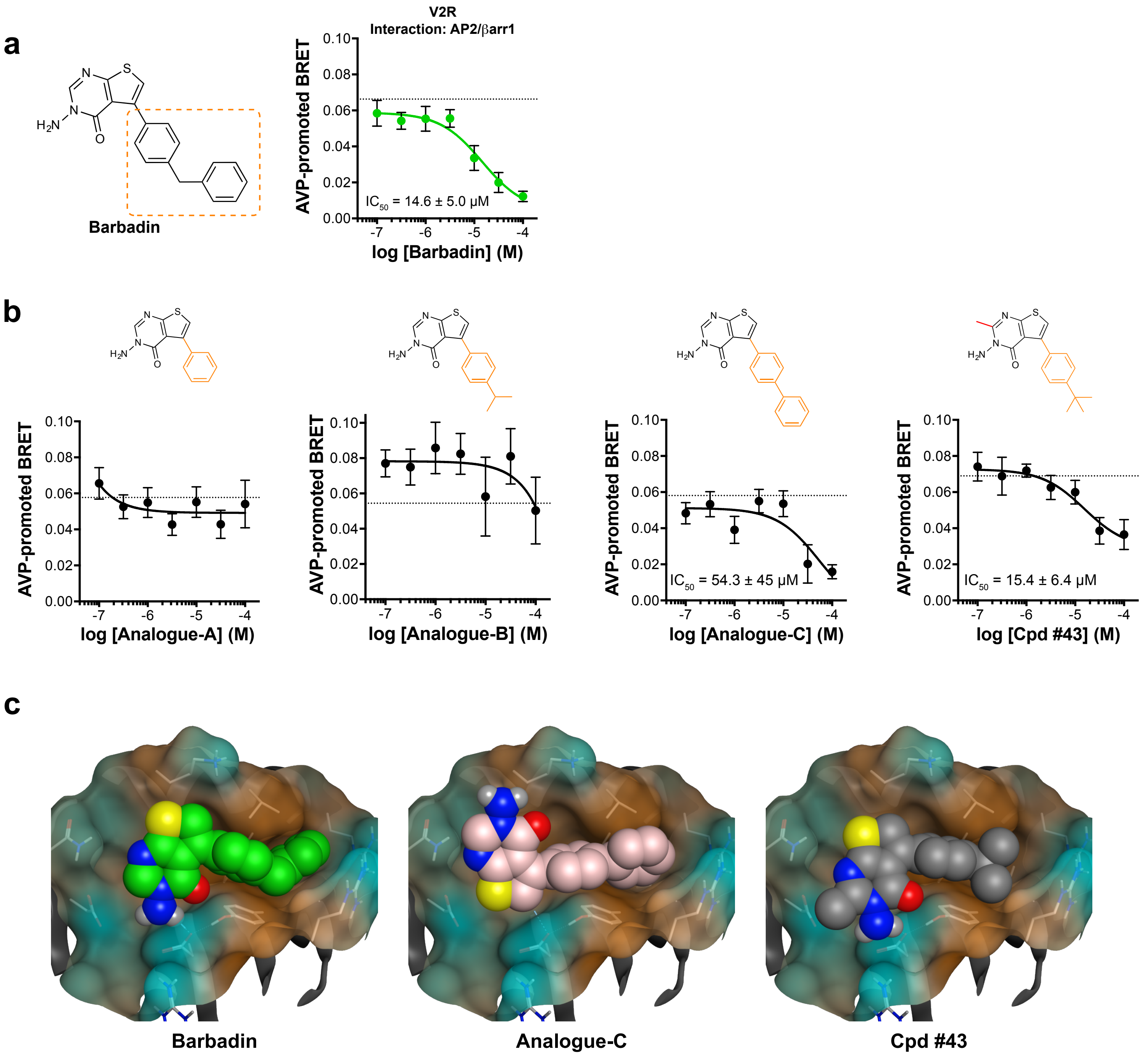

Supplementary Figure 3
Preliminary Structure-Activity-Relationship study of Barbadin.

(a,b) Concentration-response curves of Barbadin, Analogue-A, Analogue-B, Analogue-C and compound #43 on AVP-promoted (100nM) β2-adaptin/β-arrestin1 interaction. Colored substructures on Barbadin's analogues and #43 highlight the moieties where structural differences are found as compared to Barbadin's structure. Dotted line in the graphs represents the level of AVP-promoted BRET upon pre-incubation with DMSO. Data are the mean ± S.E.M. of 3 independent experiments.
(c) Docking poses of Barbadin (green), Analogue-C (pink) and compound #43 (grey) within the groove of β2-adaptin platform subdomain shown as a semi-transparent surface and colored by lipophilic potential (orange: lipophilic; cyan: hydrophilic). Beta2-adaptin residues within a 4.5 Å radius from the compounds are depicted as sticks.

Sup. Figure S4 (Bouvier)

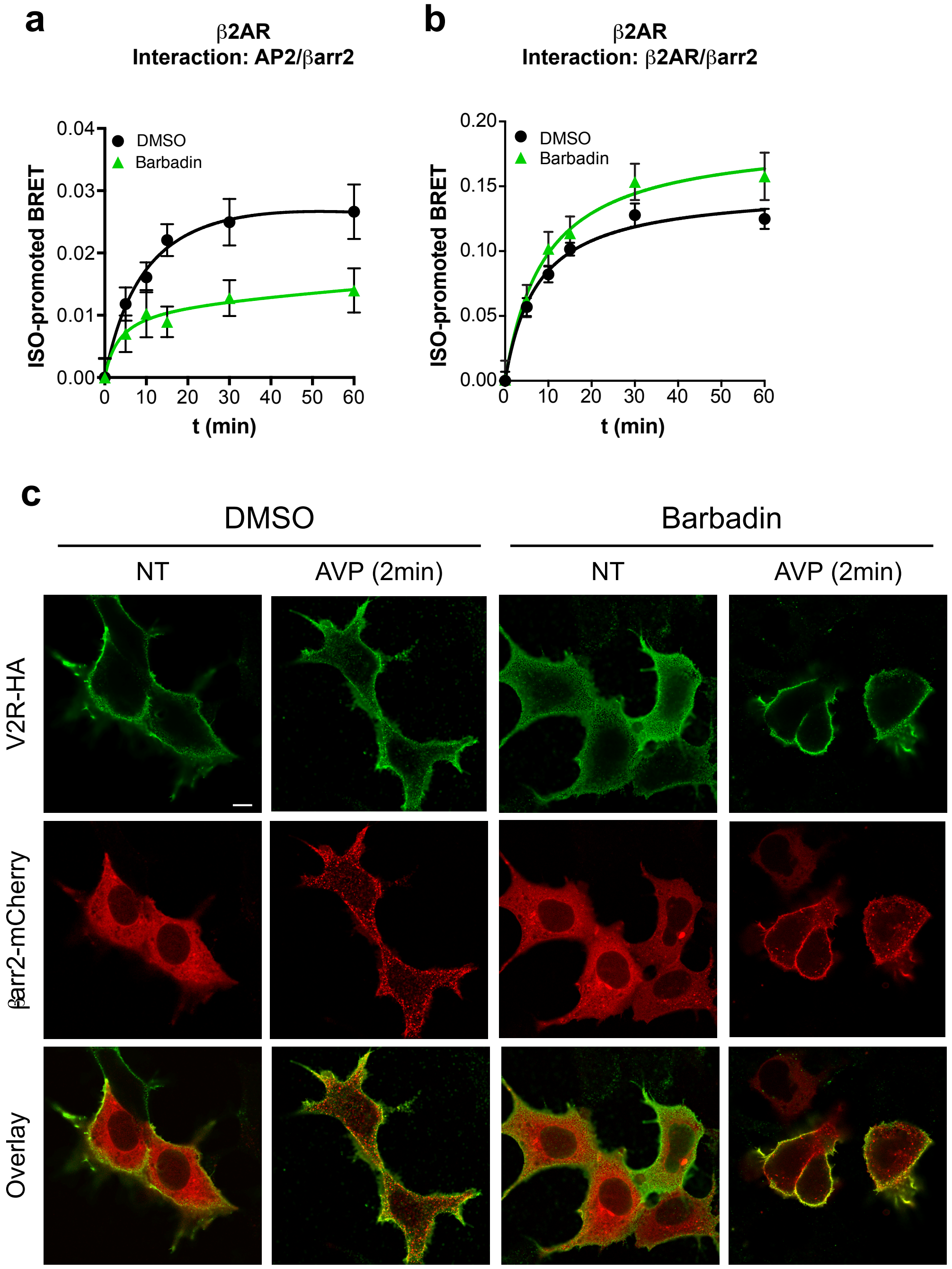

Supplementary Figure 4
Barbadin is a specific inhibitor of the interaction between β2-adaptin and β-arrestin.

(a,b) BRET-based kinetics monitoring the interaction between β-arrestin2-RlucII and β2-adaptin-YFP (a) or β 2AR-YFP (b). HEK 293T cells were pretreated with DMSO or Barbadin (100 μM) for 30 min prior to receptor stimulation with ISO (10 μM) for the indicated times. Data are the mean ± S.E.M. of 3 independent experiments.
(c) Cross-section confocal imaging of fixed HEK293 cells expressing β-arrestin2-mCherry and HA-V2R. Cells were serum-starved in DMEM for 30 min, and pre-treated with Barbadin (10 μM) for another 30 min before being stimulated with AVP (1μM) for 2 min then fixed, as described in the Methods section. Detection of V2R at the plasma membrane was performed using an antibody against HA (12CA5) in combination with a secondary antibody coupled to AlexaFluor488 (goat anti-mouse) in non-permeabilized, PFA-fixed, cells. Scale bar, 10 μm.

Sup. Figure S5 (Bouvier)

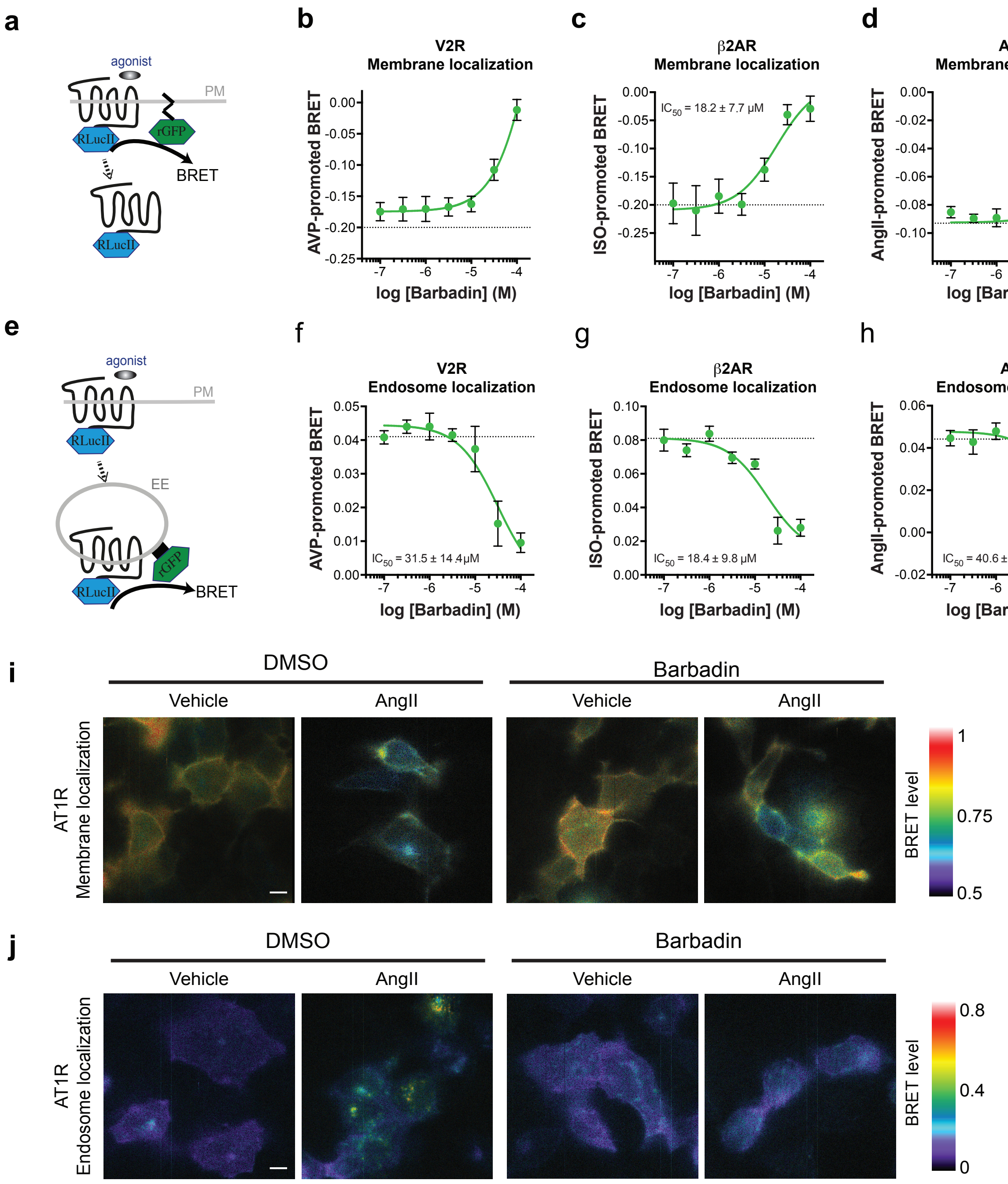

Supplementary Figure 5
Barbadin inhibits GPCRs endocytosis: ebBRET and BRET imaging.

(a,e) Schematic representation of the ebBRET-based assay used to follow agonist-induced receptor loss from the cell surface by monitoring the interaction between receptor-RLucII and rGFP-CAAX **(a)** or its translocation into endosomes using rGFP-FYVE **(e)**. PM: plasma membrane; EE: early endosome.
(b-d, f-h) V2R, β2AR or AT1R interaction with either rGFP-CAAX (**b, c** and **d**, respectively) or rGFP-FYVE (**f, g** and **h**, respectively) was assessed by BRET following HEK 293T cells pre-incubation with DMSO or Barbadin at the indicated concentrations for 30 minutes before AVP (100 nM), ISO (10 μM) or AngII (1 μM) stimulation for 30 min. Dotted line represents the level of agonist-promoted BRET upon pre-incubation with DMSO. Data are the mean ± S.E.M. of at least 3 independent experiments.
(i,j) AT1R localization imaged by BRET. HEK 293T cells were transfected with AT1R-RlucII and rGFP-CAAX **(i)** or rGFP-FYVE **(j)**, pretreated with DMSO or Barbadin (100 μM) for 30min and then stimulated with AngII (1 μM) for 30 min. To generate BRET images, the ratio of acceptor photon counts to donor photon counts was calculated for each pixel and expressed as a color-coded heat map (lowest being black and purple, and highest red and white). Scale bar, 10 μm.

Sup. Figure S6 (Bouvier)

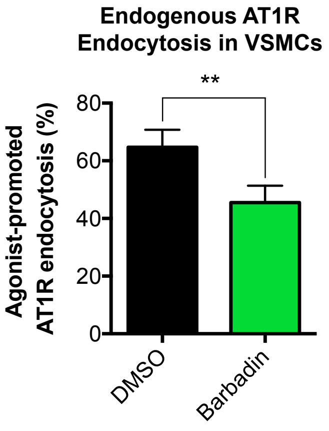

Supplementary Figure 6
Effect of Barbadin on endogenous receptor internalization in rat VSMCs.

Rat VSMCs were incubated in the absence (DMSO) or presence of Barbadin (20 µM) for 30 min at 37 °C and then subjected to [125I]-AngII binding/internalization on whole cells, as described in the Methods section. Data are expressed as percent internalization (acid-resistant binding over total binding) and represent the mean ± S.E.M. of 4 independent experiments. Statistical significance was assessed using a paired Student's t-test, comparing Barbadin-treated to vehicle (DMSO)-treated cells (**, $p < 0.01$).

Sup. Figure S7 (Bouvier)

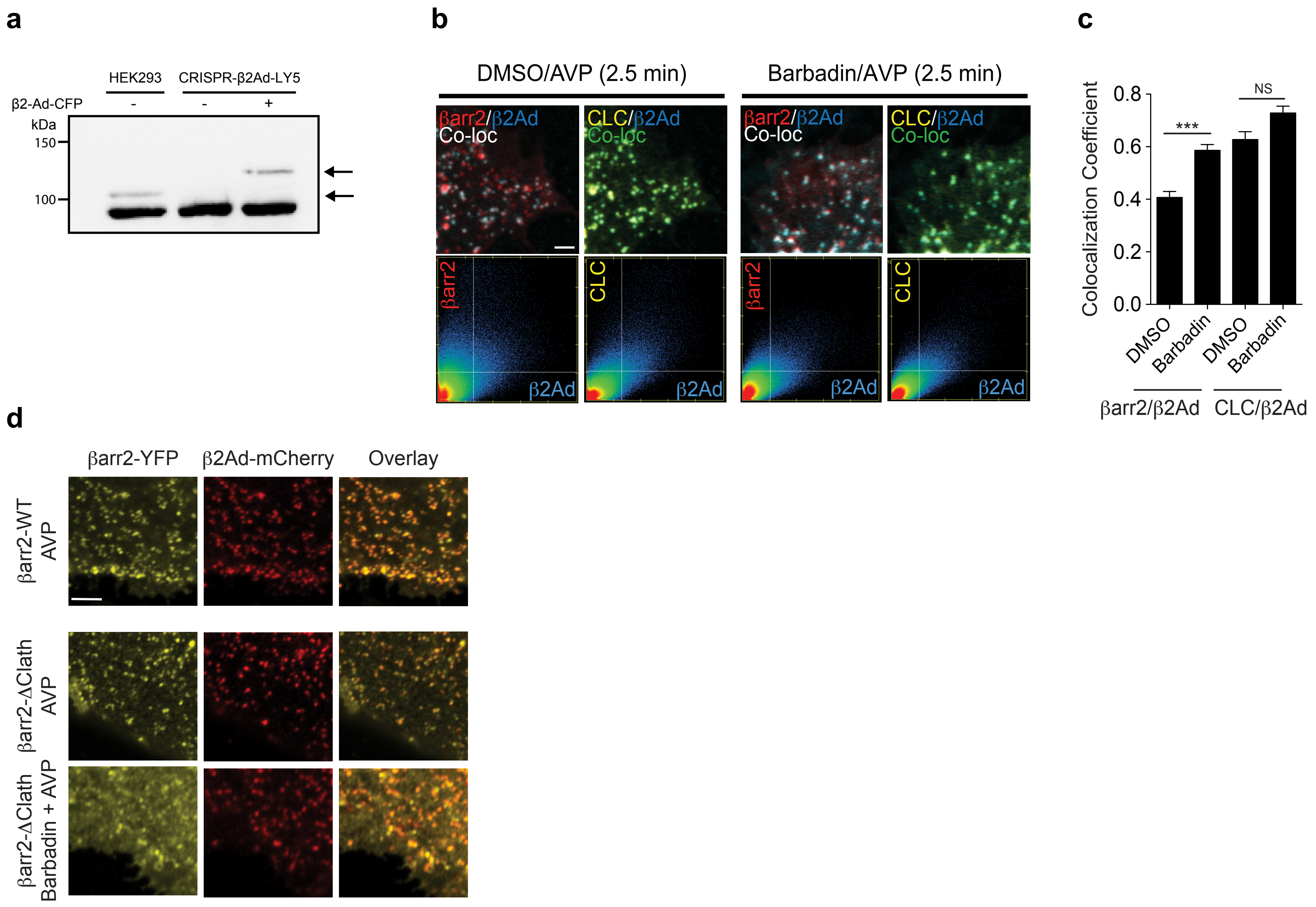

Supplementary Figure 7
Characterization of the effects of Barbadin on the colocalization of receptor/β-arrestin complexes with AP2 and clathrin in CCPs.

(a) Analysis of HEK293 cells depleted for β2-adaptin (CRISPR-β2Ad-LY5). Cells were transfected with either β 2-adaptin-CFP (β2-Ad-CFP) or pcDNA3.1 (-), and lysed 48 hours post transfection for analysis. Lysates from HEK293 and CRISPR-β2Ad-LY5 were immunoprecipitated with AP1/2 antibody, and samples analysed by Western blot using an anti-adaptin β antibody. Arrows indicate endogenous and transfected β2-adaptin.
(b,c) Visualization **(b)** and quantification **(c)** of the colocalization of either β-arrestin (βarr2) and AP2 (β2Ad) or β-arrestin and clathrin (CLC) in CRISPR-β2Ad-LY5 cells. Beta-arrestin2-mCherry and β2-adaptin-CFP, or β 2-adaptin-CFP and clathrin-YFP were transfected in HA-V2R cells. Cells were serum-starved for 30 min in the absence (DMSO) or presence of Barbadin (10 μM) and stimulated with AVP (1μM) for 2.5 min before being fixed and visualized. Colocalization of fluorescent signals is shown in top panels. Scatterplots of fluorescent signals are shown in bottom panels, and colocalization coefficients were determined from the gated signals in the scatterplots using Zen Software (Zeiss) from at least 5 cell areas from 3 different experiments. Scale bar, 2 μm. Statistical significance was assessed by one-way ANOVA, followed by a Bonferroni's multiple comparison test (*** $p < 0.001$).
(d) Colocalization of β-arrestin2 (βarr2-WT) and β-arrestin2 lacking its clathrin-binding site (βarr2-ΔClath) with AP2 (β2Ad). CRISPR-β2Ad-LY5 cells were transfected with either β-arrestin2-YFP (βarr2-WT -YFP) or β-arr2-Δ Clath-YFP, with β2-adaptin-mCherry and HA-V2R. Cells were serum-starved for 30 min, then incubated in the presence of 25 μM Barbadin for another 30 min before being subjected to a 2.5 min stimulation with AVP (1 μM). Cells were PBS-washed on ice and fixed before visualization. Scale bar, 2 μm.

Sup. Figure S8 (Bouvier)

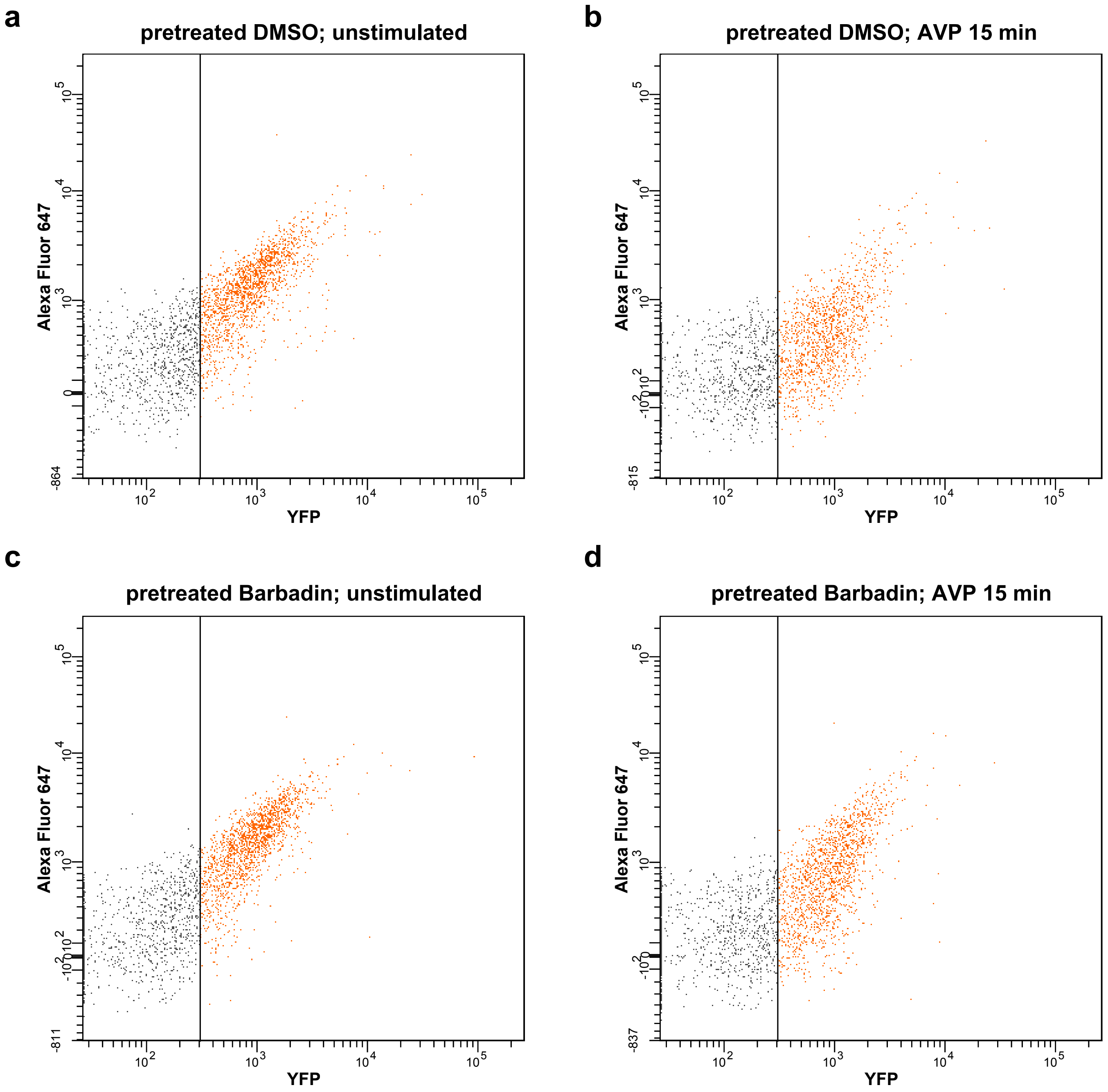

Supplementary Figure 8

Flow-cytometry assay monitoring HA-V2R-Venus expression at the cell surface (one biological replicate from *Figure 5a*)

Cells were pretreated with DMSO (**a,b**) or Barbadin (100 µM) (**c,d**) for 30 min, followed by 15 min receptor stimulation with AVP (100 nM) (**b,d**), prior to FACS analysis. The cell population (black + red dots) shown on the dot plots excludes cellular debris, dead cells and doublets. Non-transfected cells were used to define the threshold of the YFP-positive subpopulation (red dots), representing the HA-V2R-Venus expressing cells. Within this subpopulation, the plasma-membrane receptor localisation was monitored using the N-terminally fused HA-tag detected with an anti-Alexa647-coupled secondary antibody against an anti-HA antibody, in the absence of permeabilization. Then, the Alexa-647 mean values were expressed as a percentage of the "pretreated DMSO ; unstimulated" condition (set at 100%).

Sup. Figure S9 (Bouvier)

Uncropped immunoblots for Figure 3f

IP blot

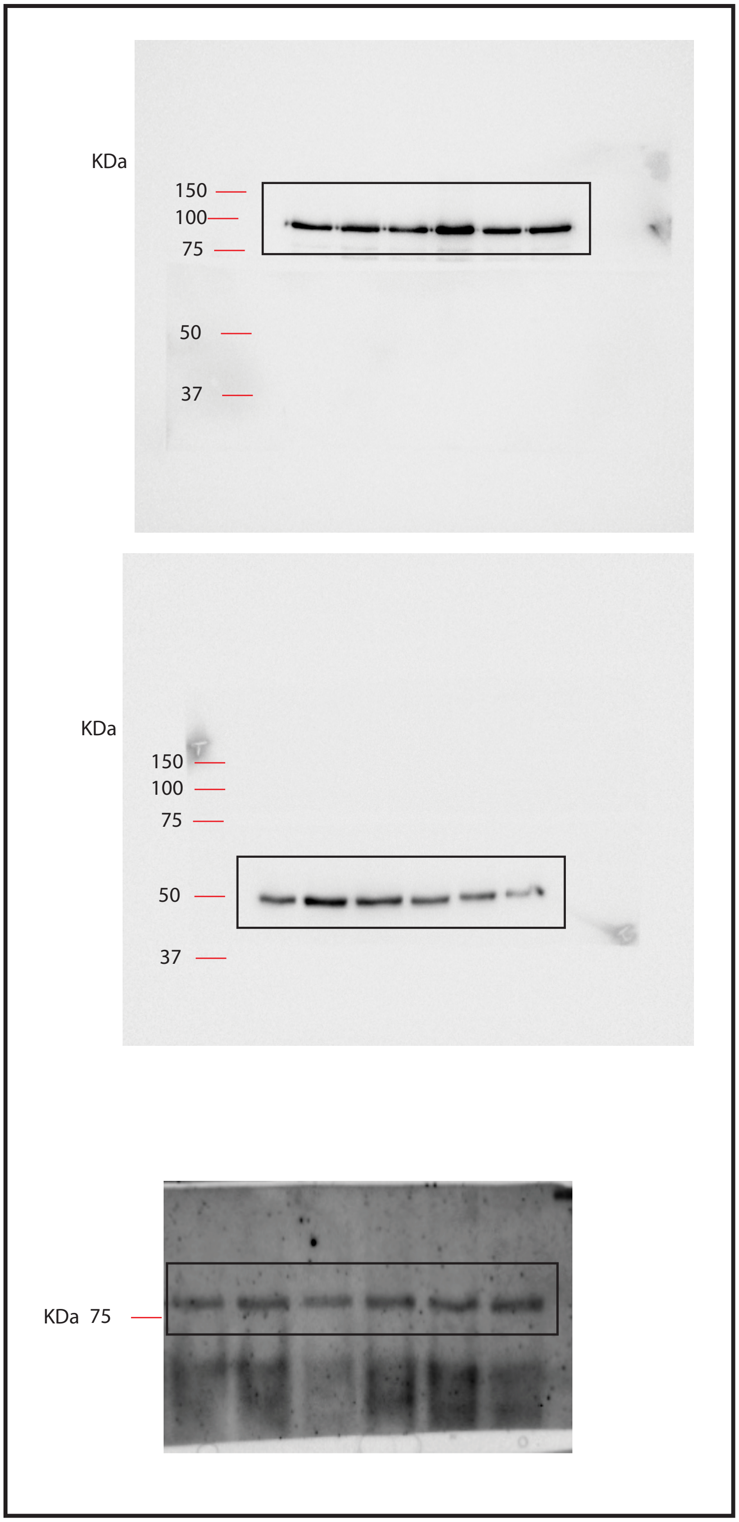

TCL blot

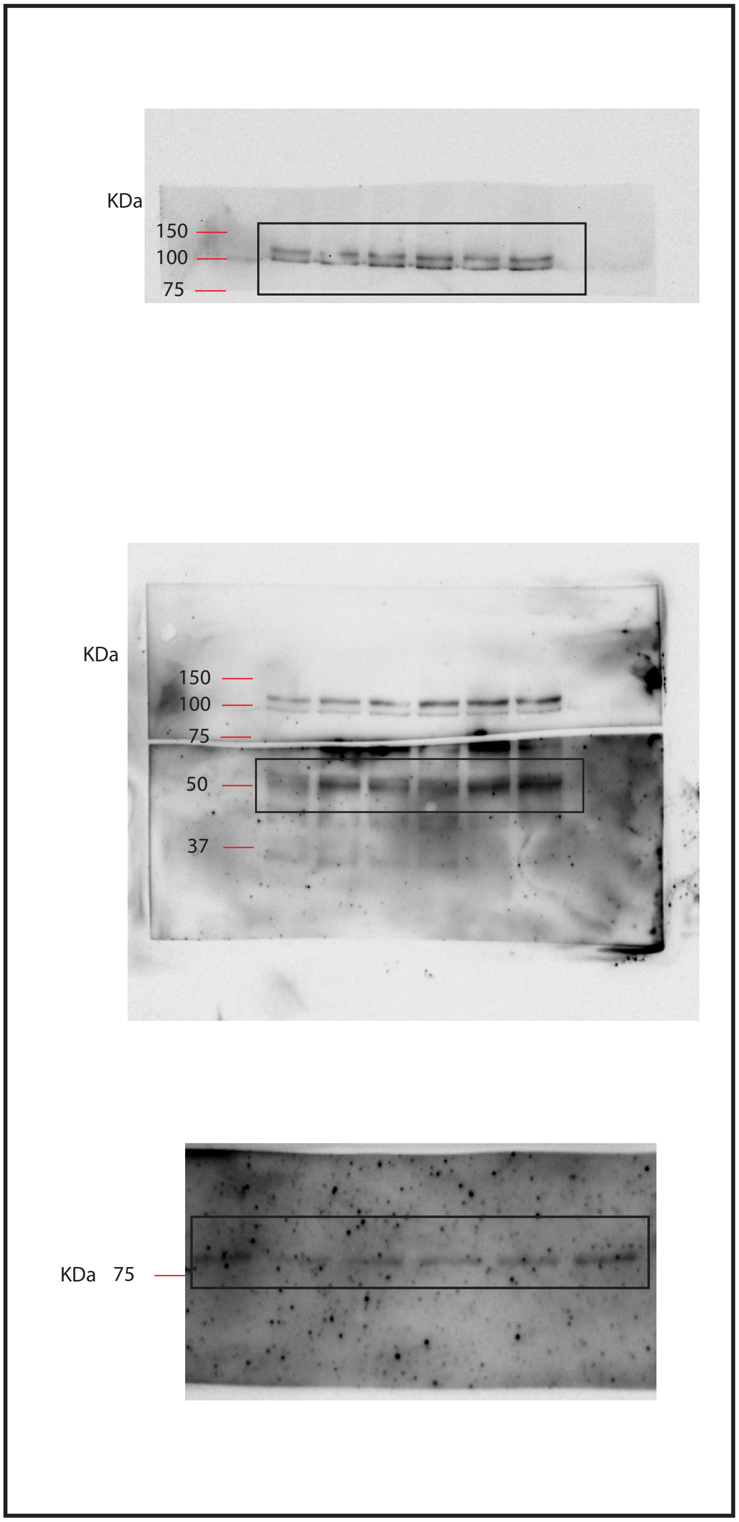

Uncropped immunoblot and Coomasie stained gel for Figure 3g

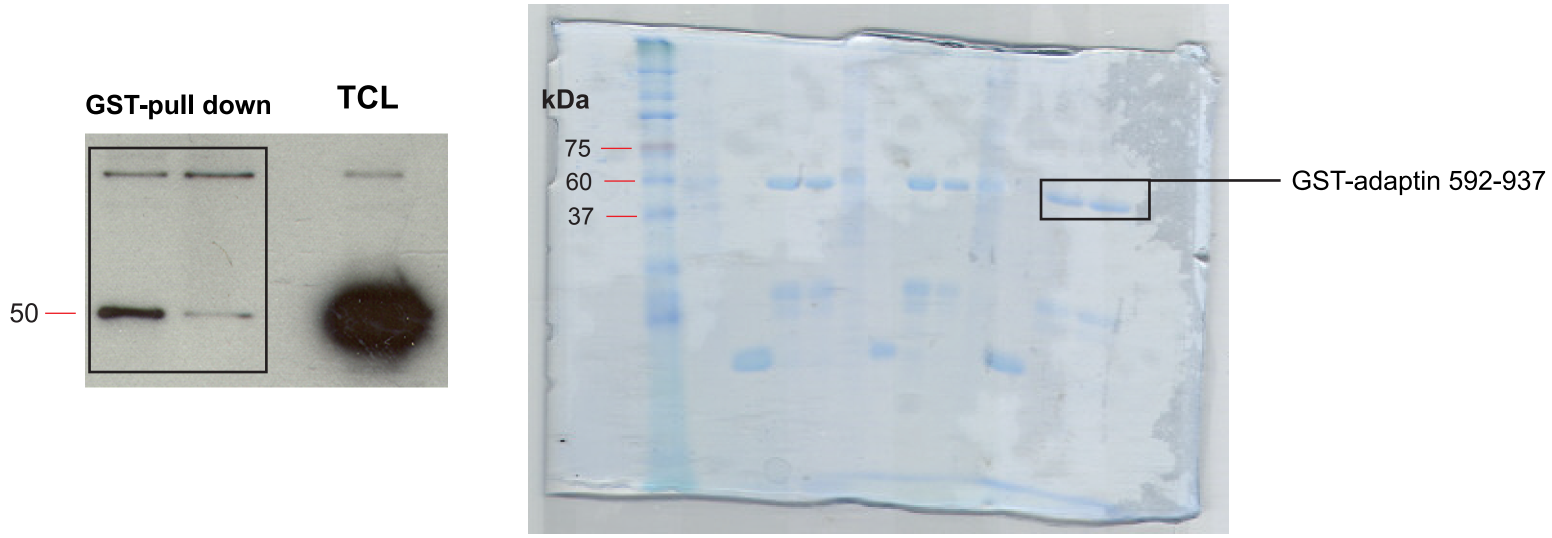

Uncropped immunoblots for Figure 7a and 7b

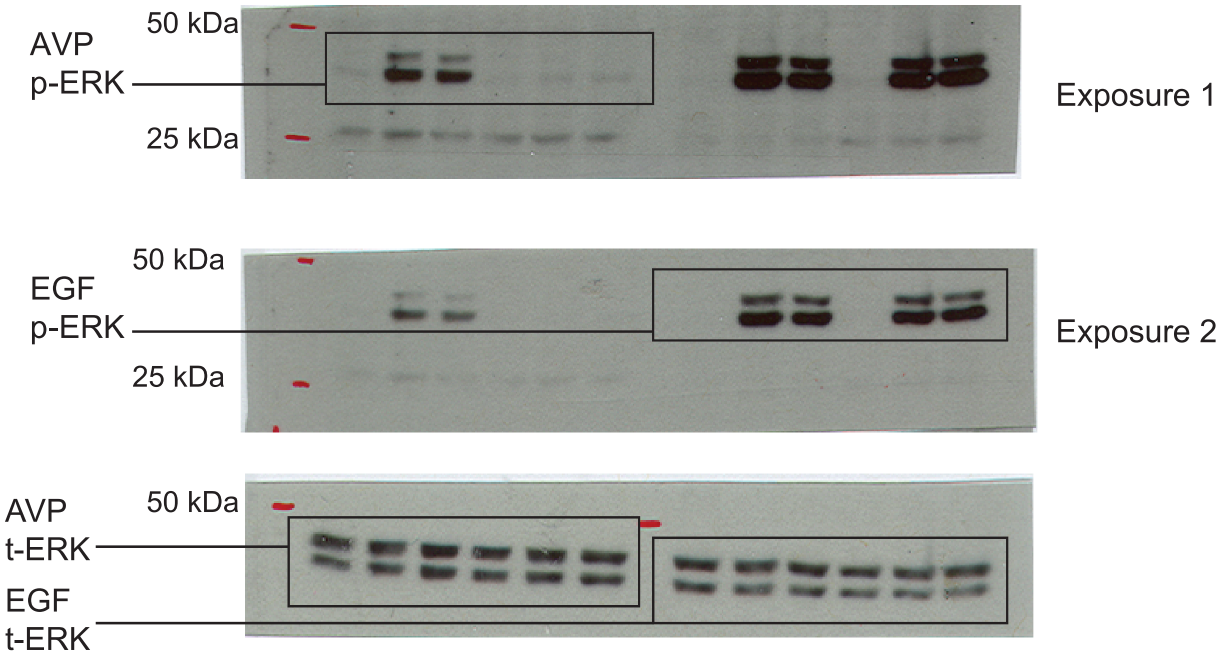

Uncropped immunoblots for Supplementary Figure 7a

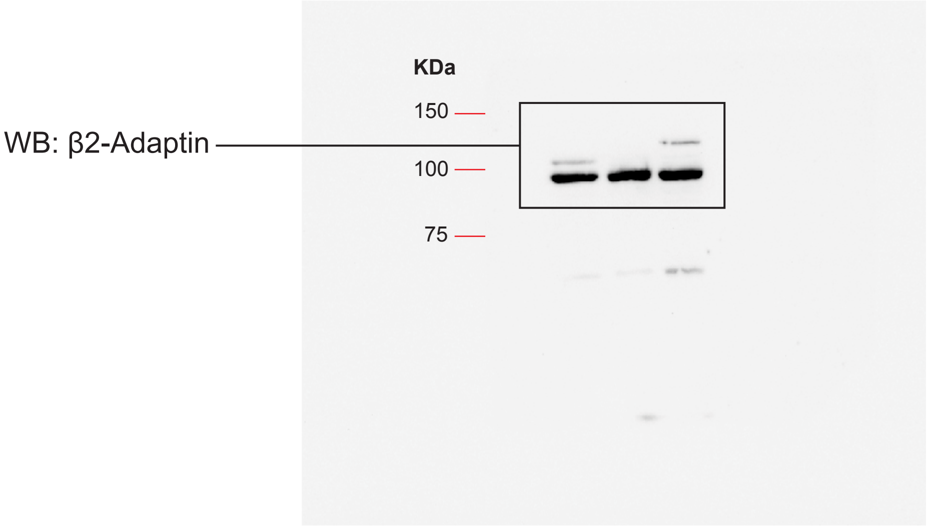

Sup. Table S1

Compound #	Sigma IDNUMBER	Compound #	Sigma IDNUMBER
**1**	L128880	**27**	R540889
**2**	L132438	**28**	R544175
**3**	L148342	**29**	R575887
**4**	L157600	**30**	R576735
**5**	L162442	**31**	R576794
**6**	L187720	**32**	R577332
**7**	L225355	**33**	R578843
**8**	L253936	**34**	R579785
**9**	L320986	**35**	R581070
**10**	L330906	**36**	R581666
**11**	L432261	**37**	R620831
**12**	R101281	**38**	R822671
**13**	R127523	**39**	R850179
**14**	R155993	**40**	R852341
**15**	R172006	**41**	R852430
**16**	R412651	**42**	R852597
**17**	R432318	**43**	R853216
**18**	R432652	**44**	R853755
**19**	R432997	**45**	R857394
**20**	R433047	**46**	R858005
**21**	R433160	**47**	R858730
**22**	R458805	**48**	R882852
**23**	R459224	**49**	R883719
**24**	R460729	**50**	R884324
**25**	R461210	**51**	R896942
**26**	R463817	**52**	R954993

Compound #	Enamine IDNUMBER
**Analogue-A**	Z56769636
**Analogue-B**	Z56821486
**Analogue-C**	Z57251039

Supplementary Table 1

List of the 52 compounds selected for the BRET-based assay screen monitoring the interaction between β 2-adaptin-YFP and β-arrestin1-RLucII, and of subsequent Barbadin's analogues. The acquisition numbers from the providers (Sigma and Enamine) are shown.
