# Supplementary material for: A new inhibitor of the β-arrestin/AP2 endocytic complex reveals interplay between GPCR internalization and signalling
Source: Nat Commun. 2017 Apr 18;8:15054. doi: 10.1038/ncomms15054 (PMC5399295; doi:10.1038/ncomms15054)
Supplement: Supplementary Dataset 1 — Amino-acid sequences of human β2-adaptin [file ncomms15054-s2.docx]

**Supplementary Data 1. Amino-acid sequences of human β2-adaptin**

**human β2 adaptin 592-937Stop (GST pull-down assay)**

HLPIHHGSTDAGDSPVGTTTATNLEQPQVIPSQGDLLGDLLNLDLGPPVNVPQVSSMQMGAVDLLGGGLDSLVGQSFIPSSVPATFAPSPTPAVVSSGLNDLFELSTGIGMAPGGYVAPKAVWLPAVKAKGLEISGTFTHRQGHIYMEMNFTNKALQHMTDFAIQFNKNSFGVIPSTPLAIHTPLMPNQSIDVSLPLNTLGPVMKMEPLNNLQVAVKNNIDVFYFSCLIPLNVLFVEDGKMERQVFLATWKDIPNENELQFQIKECHLNADTVSSKLQNNNVYTIAKRNVEGQDMLYQSLKLTNGIWILAELRIQPGNPNYTLSLKCRAPEVSQYIYQVYDSILKN-Stop

**human β2 adaptin 700-937Stop (protein thermal shift assay)**

IGMAPGGYVAPKAVWLPAVKAKGLEISGTFTHRQGHIYMEMNFTNKALQHMTDFAIQFNKNSFGVIPSTPLAIHTPLMPNQSIDVSLPLNTLGPVMKMEPLNNLQVAVKNNIDVFYFSCLIPLNVLFVEDGKMERQVFLATWKDIPNENELQFQIKECHLNADTVSSKLQNNNVYTIAKRNVEGQDMLYQSLKLTNGIWILAELRIQPGNPNYTLSLKCRAPEVSQYIYQVYDSILKN-Stop
